# Identification of biochemical indices for brown spot (*Bipolaris oryzae*) disease resistance in rice mutants and hybrids

**DOI:** 10.1371/journal.pone.0300760

**Published:** 2024-04-18

**Authors:** Areeqa Shamshad, Muhammad Rashid, Amjad Hameed, Hafiz Muhammad Imran Arshad

**Affiliations:** Nuclear Institute for Agriculture and Biology College, Pakistan Institute of Engineering and Applied Sciences NIAB-C, PIEAS, Faisalabad, Pakistan; Nuclear Science and Technology Research Institute, ISLAMIC REPUBLIC OF IRAN

## Abstract

Brown spot caused by *Bipolaris oryzae* is a major damaging fungal disease of rice which can decrease the yield and value of produce due to grain discoloration. The objectives of the current study were to investigate and understand the biochemical indices of brown spot disease resistance in rice. A total of 108 genotypes (mutant and hybrid) along with Super Basmati and parent RICF-160 were evaluated against brown spot disease. The genotypes exhibiting resistant and susceptible responses to brown spot disease according to the IRRI standard disease rating scale were screened and selected. To study the biochemical response mechanism, forty five selected genotypes along with Super Basmati and RICF-160 were analyzed using the biochemical markers. The physiological and biochemical analysis provided valuable insights and confirmed the resistance of rice hybrids and mutants against brown spot disease. Positive correlations were observed among stress bio-markers and disease response. Rice genotypes i.e. Mu-AS-8, Mu-AS-19, Mu-AS-20 and Mu-AS-35 exhibited moderate resistant response while Hy-AS-92, Hy-AS-98, Hy-AS-99, Hy-AS-101, Hy-AS-102 and Hy-AS-107 showed resistant response to brown spot disease. Brown spot resistant rice genotypes had lesser values of malondialdehyde and total oxidant status and higher antioxidant activities i.e. superoxide dismutase, peroxidase, total phenolic content and lycopene. The selected resistant rice genotypes had resistance capacity against *Bipolaris oryzae* stress. In conclusion, identified resistant mutants i.e. Mu-AS-8, Mu-AS-19, Mu-AS-20 and Mu-AS-35 and hybrids i.e. Hy-AS-92, Hy-AS-98, Hy-AS-99, Hy-AS-101, Hy-AS-102 and Hy-AS-107 could be used in rice breeding program to achieve sustainable rice production by coping the emerging challenge of brown spot disease under variable climate conditions.

## Introduction

Rice (*Oryza sativa* L.) is a staple food crop of approximately half of the world’s population (International Rice Research Institute; *IRRI*, *2022)* [1]. About 1/3^rd^ of the world’s rice cultivating area lies in Pakistan. It is 2^nd^ staple cereal crop after wheat, 2^nd^ cash crop after cotton and secured 11^th^ position in rice production and 5^th^ as an exporter [2–5]. During 2020–21 in Pakistan, the crop was cultivated on 3,335 thousand hectares, reflecting an increase 9.9 percent as compared to last year’s sown area of 3,034 thousand hectares. Pakistan economy survey witnessed a record production growth of 13.6 percent to 8.419 million tonnes against 7.414 million tonnes as compared to last year. The demand for rice increases with the increasing population and is expected to rise further by 38% within the next 30 years [6].

Super Basmati is a special variety of rice with extra-long, slender grain and is renowned across the world for its distinct aroma [7]. This variety since its inception in 1997 is still in the field due to its wider adaptability and currently covers about 40% share in Basmati rice area. Basmati rice has high quality attributes and narrow genetic base. Under variable climate conditions, new pathogens or new races of old pathogens are emerging which makes the cultivated rice varieties susceptible [8–10]. Among the important diseases of rice, Brown spot is one of the major damaging rice fungal diseases in the world which is caused by several fungi, among which anamorph *Bipolaris oryzae* is particularly important.

Among the top ten fungal diseases, Blast is the leading one causing severe damage in Africa but brown spot is a potent yield reducer amongst the fungal rice diseases in Asia. From the export point of view, this pathogen causes quantity and quality losses that are associated with the disease incidence on the leaves and grain and causes kernel discoloration, which affects the drying, shelling, milling and processing of the rice due to weight loss [11–14]. It becomes more important not only because of quality deterioration of grains but also due to brown spots on grain which may lead to rejection of consignments at international market. Because, for rice consumers, whole grains free from defects are preferred and this factor determines the price that growers will receive [15]. Yield losses due to brown spot disease on grains have been recorded in the range of 16% to 43%. Yield losses due to infection of brown spot on rice leaves in comparison with infection of glumes needed to be investigated. Brown spot is currently regarded as a serious rice disease worldwide [13, 16]. To check the resistance of different rice cultivars to brown spot disease, several experiments have been carried out [17].

Therefore, the present study was planned with the following main objectives: i) identify rice genotypes resistant to brown spot disease at the seedling stage in the nursery (ii) assess the effect of the *B*. *oryzae* inoculum pressure on the biochemistry of mutant/hybrid lines of rice at the seedling stage. New basmati rice varieties with resistance to brown spot disease were also developed using modern molecular breeding techniques. These days, one of the main research focuses in basmati research and development of the basmati mutants and hybrids. Moreover, the use of resistant varieties is the best and reasonable solution to control the brown spot of rice. Manangement of brown spot disease is the task to enhance the production of rice. Breeding of stress-resistant crops is the most efficient strategy to maintain yield in stress-prone marginal land. To create genetic variability through conventional breeding approaches, rice quality is deteriorated. Mutation breeding coupled with other disciplines provides an excellent platform for the improvement of one or two desirable traits without compromising quality attributes. Recently, rice mutant varieties (874) have been released using mutation breeding approaches. Moreover, the mutants are non-genetically modified organisms (Non-GMOs). It is thus important to identify genetic resources with high resistance to biotic stress using nuclear techniques. The genetic improvement of this food crop can serve as a major component of sustainable food production.

## Material and method

### Plant material and study area

Rice germplasm consists of stable mutant lines and hybrids. The mutant genotypes were developed by irradiating parent line RICF-160 with different physical mutagen (^137^Cs) doses (150 Gy, 200 Gy and 250 Gy) and hybridized material was developed by attempting cross RICF-160 /ELD at Nuclear Institute for Agriculture and Biology (NIAB), Faisalabad. A total of 108 genotypes along with check variety (Super Basmati) were evaluated against brown spot disease following the *IRRI*, [18] *Philippines (2014)* **(S1 Table)**.

The pathological experimental field area of NIAB was located within a farmer’s field and managed by the farmer and farmer practices. Mean rainfall in the study area ranges between 300–350 mm annually, whereas mean monthly temperature goes as high as 48°C in June. Recommended cultural practices were followed for raising of rice seedlings. No obvious standing water, weeds, diseases, or insect pests were observed at any of the sites during the growing season of rice. Before experimentation, soil samples were collected from 0–15 cm soil depth and were air-dried, ground to pass through a 2 mm sieve for analysis of different physicochemical properties (Soil texture, organic matter, nitrogen, phosphorus and potassium). The experimental soil was clay loamy, organic matter (0.9%) and plant-available nutrients; i.e. nitrogen (9 mg NO_3_ kg), P (12.33 mg kg^−1^) and K (120 mg kg^−1^).

### Brown spot disease screening

The response of 108 rice genotypes along with Super Basmati and parent RICF-160 to disease (brown spot) was evaluated under brown spot nursery following the IRRI, SES, Philippines method of disease screening in the field. Germplasm seeds were sown on a raised bed of 30 cm in a single row and after two test rows; a highly susceptible variety (Basmati 2000) was sown in a single row to aid in the spread of the disease. With 10 cm between the rows, three replications of each test genotype were seeded. Additionally, two parallel rows of the disease-susceptible spreader species were planted all around the nursery. Three times daily overhead irrigation and a thin layer of farm yard manure were used to cover the seeds. The test plants were treated with *B*. *oryzae* inoculum after three weeks [19]. In laboratory conditions, the fungus was sub-cultured on PDA plates for inoculum preparation. For mass multiplication of the fungus (*B*. *oryzae*), One kg of seeds of susceptible rice variety (Basmati-2000) were put in four polythene bags (250 g) each, moisturized with distilled water. Polythene bags were plugged with cotton plug to tighten the top of the polythene bag and then autoclaved. Seven day old fungal cultures (5 mm disc) were placed in seed bags and incubated for 15 days at room temperature. After that, 250 ml of autoclaved distilled water was added to seed bags and thoroughly stirred while wearing gloves and the resulting suspension was filtered through muslin fabric and placed in a beaker to make the require volume for inoculation (19). The inoculum concentration (1 × 10^5^ spores/ml) was measured using a heamocytometer [20] and about 25 ml of spore suspension was sprayed on each 30 cm row of rice test line by hand sprayer. According to the *IRRI* (2014) standard disease rating scale, disease data were obtained after 3 weeks **(Table 1)**. Ten plants from each genotype were examined for disease assessment, and statistical analysis was performed using the mean.

**Table 1:** Disease scoring scale of brown spot in rice.

Disease Scale	Infection	Host behavior	Symbol
0	No incidence	Immune	I
1	1–5%	Resistant	R
2	6–15%	Moderately resistant	MR
3	16–25%	Moderately Susceptible	MS
4	26–50%	Susceptible	S
5	>50%	Highly susceptible	HS

As described by IRRI (2014).

### Biochemical analysis

In order to examine the biochemical responses of plants to brown spot disease under five level of disease i.e, resistance (R), moderate resistance (MR), moderate susceptible (MS), susceptible (S), and highly susceptible (HS). The genotypes having 6 resistant phenotypes and 16, 4, 8 and 9 genotypes having MR, MS, S and HS phenotypes respectively. The leaf samples (15–20 leaves) were collected after three weeks of inoculation from the disease screening nursery. Selected the sample number on the basis of disease percentage as given in **Table 1** and stored at -1°C for analysis. The biochemical behavior of the selected 45 genotypes 6 (R) genotypes, 16 (MR), 4 (MS), 8 (S) and 9 (HS) along with Super Basmati and parent (RICF-160) to the stress was confirmed by using the biochemical markers including enzymatic, non-enzymatic, hydrolytic antioxidants, and certain other biochemical assays were performed at Marker Assisted Breeding lab-1, Plant Breeding and Genetics Division, NIAB, Faisalabad, Pakistan **(S2 Table)**.

### Extraction for estimating the antioxidant enzyme activities

Fresh leaves of plants 0.1 g were sampled. To preserve the samples were macerated using 1 mL of 50 mM potassium phosphate buffer (pH 7.8). All of the samples were vortexed before being centrifuged at 15,000 g for 10 minutes at 4°C to homogenize the mixture. Enzymatic, non-enzymatic, hydrolytic antioxidants and other biochemical parameters were measured in the supernatant [21]. These analyses were performed in three replicates for all biochemical parameters.

#### Enzymatic antioxidant activities

*Superoxide dismutase activity (SOD)*. Rice leaves were homogenised in a solution containing 50 mM potassium phosphate buffer (pH 7.0), 0.1 mM EDTA, and 1 mM dithiothreitol (DTT) to determine SOD activity following the previous approach as described in [22]. The activity of SOD was assayed by measuring its ability to inhibit the photochemical reduction of nitroblue tetrazolium (NBT). The amount of enzyme that inhibited the photochemical degradation of NBT by 50% was determined as one unit of SOD activity.

*Ascorbate peroxidase activity (APX)*. Rice leaves were homogenized in 50 mM potassium phosphate buffer (pH 7.0) to measure the APX activity [23]. The assay solution included an assay buffer containing 10 mM ascorbic acid, 0.5 M EDTA, and 200 mM potassium phosphate buffer, H_2_O_2_ (1 mL), and supernatant 50 μL for APX measurement. The oxidation rate of ascorbic acid was estimated by following the decrease in absorbance at 290 nm after every 30 second to a minute.

*Peroxidase activity (POD)*. Rice leaves were homogenized in a solution containing 50 mM potassium phosphate buffer (pH 7.0), 0.1 mM EDTA, and 1 mM DTT to determine POD activity. The activity of POD was assessed using the method of [24] with some modification. For measurement of POD activity, the assay solution contained distilled water (545 μl), 200 mM phosphate buffer (pH 7.0), 200 mM guaiacol, 400 mM H_2_O_2_, and 15 μl enzyme extract. The reaction was initiated after adding the enzyme extract. Every 20 seconds to a minute, the absorbance of the reaction solution at 470 nm (UV-VIS spectrophotometer U-2800) increased. One unit of POD activity was defined as an absorbance change of 0.01 min^−1^. Enzyme activity was expressed on the leaf weight basis.

*Catalase activity (CAT)*. CAT was estimated by homogenizing rice leaves in a solution containing 50 mM potassium phosphate buffer (pH 7.0) and 1 mM dithiothreitol (DTT) by following the method [25]. For measurement of CAT activity, the assay solution contained 50 mM phosphate buffer (pH 7.0), 59 mM H_2_O_2_, and 100 μl enzyme extract. The decrease in absorbance of the reaction solution was recorded at 240 nm after every 20 s to a minute by UV-VIS spectrophotometer (U-2800). An absorbance change of 0.01 min^−1^ was defined as 1 U of CAT activity. Enzyme activity was expressed on leaf weight basis.

#### Non-enzymatic antioxidants

*Total Phenolic Contents (TPC)*. The TPC in rice leaves was determined using the Folin-Ciocalteu (F-C) reagent and a micro colorimetric approach [26]. Leaf samples (0.5 g) were homogenized in 500 μl of ice-cold 95% methanol using an ice-cold mortar and pestle. The samples were left to incubate for 48 hours at room temperature in the dark. After that, the samples were centrifuged at 14,462×g for 5 min at room temperature. For the purpose of measuring TPC, the supernatant was taken off. After extensively vortexing 100 μl of supernatant with 100 μl of 10% (v/v) F-C reagent, 800 μl of 700 mM Na_2_CO_3_ was added. After that, samples were kept at room temperature for one hour. Blank absorbance was measured at 765 nm by UV-VIS spectrophotometer. Using varied concentrations of gallic acid, a standard curve was created and a linear regression equation was derived. The linear regression equation was used to determine the phenolic content of samples.

*Tannins*. The determination of tannins 0.1 g of PVP was added to the above assay (TPC) and vortex until mixed properly. It was centrifuged at 14000 rpm for 10 minutes. Absorbance was recorded at 765 nm.

*Ascorbic acid (AsA)*. The previously reported [27] 2,6- dichloroindophenol (DCIP) method was used to determine reduced AsA. Vitamin C converts a molecule of DCIP into DCIPH_2_, which is identified by a decline in absorbance at 520 nm in this approach. A standard curve was created using a series of known ascorbic acid concentrations, and the ascorbate concentration in unknown samples was determined using a simple linear regression equation.

*Total flavonoid content (TFC)*. The aluminum chloride colorimetric method was used to determine the total flavonoid concentration [28]. The sample (400 μL + 1600 μL dH_2_O) was mixed with 0.1 mL of 10% aluminum chloride hexahydrate, 0.1 mL of 1 M potassium acetate, and 2.8 mL of deionized water. The absorbance of the reaction mixture was measured using a UV-VIS spectrophotometer at 415 nm after a 40-minute incubation at room temperature.

#### Hydrolytic enzymes

*Esterase activity*. The activity of α-esterases and β-esterases was determined using a reported method [29]. Substrates included α -naphthyl acetate and β-naphthyl acetate. A substrate solution [30 MMA or b-naphthyl acetate, 0.04 M phosphate buffer (pH 7) and 1 percent acetone] as well as enzyme extract, were included in the reaction mixture. The mixture was placed for 15 minutes at 27°C in the dark, and then 1 mL of staining solution (1 percent Fast blue BB and 5 percent SDS mixed in a 2:5 ratio) was added and incubated for 20 minutes at 27°C in the dark. The absorbance at 590 nm was used to calculate the amount of α-and β-naphthol produced in μM min^−1^g^-1^ leaf weight.

*Protease activity*. The leaf samples were homogenized in a medium containing 50 mM potassium phosphate buffer to determine the protease activity (pH 7.8) [30]. Casein digestion experiment was used to assess protease activity. By this method, one unit represents the quantity of enzyme that releases acid soluble fragments equivalent to 0.001 A280 min^-1^ at 37°C and pH 7.8.

*Alpha-amylase activity*. For the assessment of rice leaves alpha-amylase activity, an earlier described method with certain modifications [21] was followed.

#### Other biochemical parameters

*Total oxidant status (TOS)*. TOS was determined using a defined method [31], which is based on the oxidation of ferrous ion to ferric ion by oxidants present in the sample in an acidic medium and the measurement of ferric ion by xylenol orange [32]. Sample extract, reagent R1, and reagent R2 were all included in the test combination. A UV-VIS spectrophotometer was used to detect absorption at 560 nm after 5 minutes. A standard curve was drawn using hydrogen peroxide. The results were expressed in μM H_2_O_2_ equivalent per L.

*Malondialdehyde content (MDA)*. The level of lipid peroxidation in the leaf tissue was tested using [33] method, which used the thiobarbituric acid (TBA) reaction to quantify the quantity of MDA. The homogenate was centrifuged at 14,462 ×g for 5 min. The mixture was heated to 95°C for 30 minutes and then quickly cooled in an ice bath. The UV-VIS spectrophotometer was used to determine the absorbance of the supernatant at 532 nm after centrifugation at 14,462 ×g for 10 minutes, and the result for non-specific absorption at 600 nm was subtracted. The MDA was calculated by using an extinction coefficient of 155 mM^−1^ cm^−1^.

*Total soluble proteins (TSP)*. For TSP determination in rice leaves, supernatant (5 μL) of sample extract and 0.1 N NaCl were mixed with 1.0 mL of dye reagent (100 mg). Quantitative protein determination was done by using the Bradford method. This mixture was kept for 5 min to form protein dye complex before taking readings. Blank-corrected reading was calculated at 595 nm by a UV-VIS spectrophotometer [34].

*Pigment analysis*. The pigment contents (lycopene, chlorophyll a, chlorophyll b, total chlorophyll and carotenoids) in the leaves were determined by the given method [35]. Absorbance was measured at 663, 645, 505, 453, and 470 nm wavelengths using a using UV-VIS spectrophotometer (U-2800).

### Physiological parameter

Physiological trait like photosynthesis rate was measured after three weeks of *B*. *oryzae* inoculation with the help of a LI-1600 Steady State porometer. Data was recorded from ten plants from each genotypes after early sunrise and then calculated using the formula [36].

The photosynthetic rate (μmolm^−2^s^−1^) was computed using the formula:

$$\text{Photosynthetic}\;\text{rate}=\text{Sc}\;\left(\text{mmol}\;m^{-2}s^{-1}\right)/\mathrm{Tr}\;\left(\mu g\;cm^{-2}s^{-1}\right)\times 10$$

Sc = stomatal conductance; Tr = transpiration rate.

### Statistical analysis

Statistical analysis was performed by using R studio version 3.6.2. For text mining the ‘tm’ package was used. For principle component analysis, the packages FactomineR and Facto extra were used. Data were subjected to cluster analysis and correlation to identify the association among traits using p heat map and corrplot packages. Data were presented in the graphs as mean values, standard error and least significant difference (*LSD*). The collected data analysis for all parameters was based on the analysis of variance (ANOVA) to tests for significance.

## Results

### Resistance level of rice genotypes

**Fig 1:**
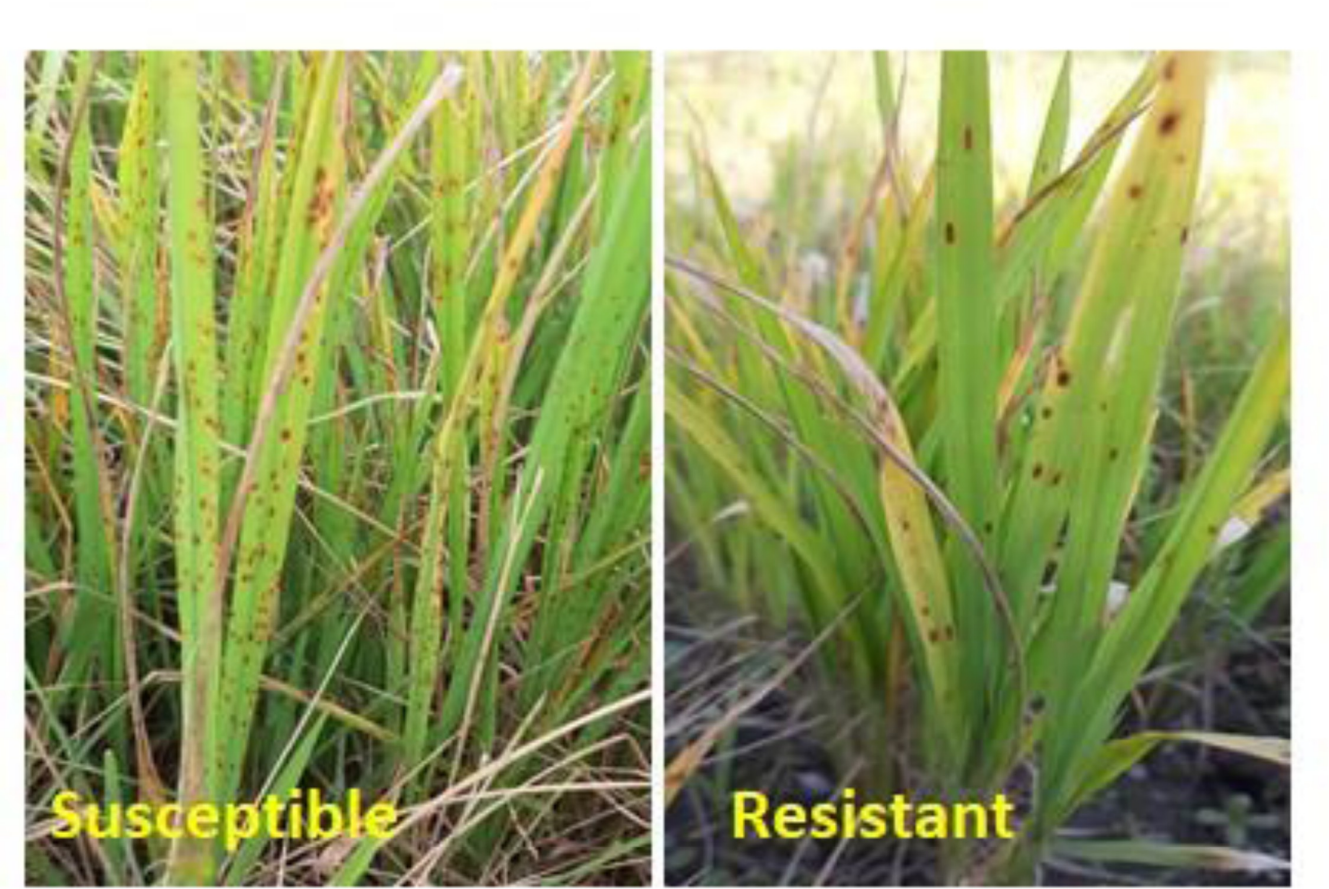
*Bipolaris oryzae* the causal organism of brown spot disease in rice. The disease symptoms are shown in susceptible and resistant rice genotypes, typical lesions appeared as dark brown in color and typical yellow halo around the spot on susceptible rice leaves.

The results showed that there was a significant difference among rice germplasm in terms of disease damage (%) (P≤0.05). Disease (%) mean values were highly variable due to differences in the response of genotypes to the pathogen. Mean values of disease damage (%) categorized rice genotypes into five groups namely; resistant (R), moderately resistant (MR), moderately susceptible (MS), susceptible (S) and highly susceptible (HS) see **Table 1**. The genotypes Mu-AS-47, Hy-AS-50, Hy-AS-55 and Super Basmati showed the highest disease (%) mean value and they were regarded as susceptible genotypes. While, Mu-AS-20, Mu-AS-35 exhibited moderately resistant responses. The Hy-AS-92, Hy-AS-98 and Hy-AS-101 showed the lowest mean damage (%) values and were selected as resistant genotypes while the parent (RICF-160) falls in the moderate susceptible category **(Table 2)**. The typical symptoms of susceptible and resistant genotypes are shown in **Fig 1.** None of the tested genotypes was immune according to the standard disease rating scale given by IRRI, 2014.

**Table 2:** Phenotypic response of diverse rice lines against brown spot disease.

Sr.No.	Genotype	D (%)	Response	Sr. No	Genotype	D (%)	Response
1	Mu-AS-1	27	S	55	Hy-AS-55	38	S
2	Mu-AS-2	7	MR	56	Hy-AS-56	85	HS
3	Mu-AS-3	75	HS	57	Hy-AS-57	15	MR
4	Mu-AS-4	20	MS	58	Hy-AS-58	85	HS
5	Mu-AS-5	35	S	59	Hy-AS-59	82	HS
6	Mu-AS-6	40	S	60	Hy-AS-60	75	HS
7	Mu-AS-7	17	MS	61	Hy-AS-61	90	HS
8	Mu-AS-8	10	MR	62	Hy-AS-62	78	HS
9	Mu-AS-9	5	R	63	Hy-AS-63	60	HS
10	Mu-AS-10	58	HS	64	Hy-AS-64	92	HS
11	Mu-AS-11	27	S	65	Hy-AS-65	20	MS
12	Mu-AS-12	50	S	66	Hy-AS-66	60	HS
13	Mu-AS-13	17	MS	67	Hy-AS-67	40	S
14	Mu-AS-14	22	MS	68	Hy-AS-68	18	MS
15	Mu-AS-15	58	HS	69	Hy-AS-69	25	MS
16	Mu-AS-16	18	MS	70	Hy-AS-70	32	S
17	Mu-AS-17	10	MR	71	Hy-AS-71	10	MR
18	Mu-AS-18	18	MS	72	Hy-AS-72	17	MS
19	Mu-AS-19	13	MR	73	Hy-AS-73	35	S
20	Mu-AS-20	13	MR	74	Hy-AS-74	35	S
21	Mu-AS-21	33	S	75	Hy-AS-75	18	MS
22	Mu-AS-22	7	MR	76	Hy-AS-76	7	MR
23	Mu-AS-23	22	MS	77	Hy-AS-77	13	MR
24	Mu-AS-24	22	MS	78	Hy-AS-78	8	MR
25	Mu-AS-25	30	S	79	Hy-AS-79	7	MR
26	Mu-AS-26	22	MS	80	Hy-AS-80	28	S
27	Mu-AS-27	17	MS	81	Hy-AS-81	7	MR
28	Mu-AS-28	8	MR	82	Hy-AS-82	5	R
29	Mu-AS-29	18	MS	83	Hy-AS-83	7	MR
30	Mu-AS-30	8	MR	84	Hy-AS-84	3	R
31	Mu-AS-31	5	R	85	Hy-AS-85	7	MR
32	Mu-AS-32	13	MR	86	Hy-AS-86	17	MS
33	Mu-AS-33	17	MS	87	Hy-AS-87	10	MR
34	Mu-AS-34	23	MS	88	Hy-AS-88	3	R
35	Mu-AS-35	13	MR	89	Hy-AS-89	8	MR
36	Mu-AS-36	17	MS	90	Hy-AS-90	3	R
37	Mu-AS-37	7	MR	91	Hy-AS-91	3	R
38	Mu-AS-38	30	S	92	Hy-AS-92	2	R
39	Mu-AS-39	10	MR	93	Hy-AS-93	2	R
40	Mu-AS-40	7	HS	94	Hy-AS-94	7	MR
41	Mu-AS-41	55	MR	95	Hy-AS-95	3	R
42	Mu-AS-42	13	MR	96	Hy-AS-96	2	R
43	Mu-AS-43	17	MS	97	Hy-AS-97	5	R
44	Mu-AS-44	15	MR	98	Hy-AS-98	2	R
45	Mu-AS-45	7	MR	99	Hy-AS-99	2	R
46	Mu-AS-46	20	MS	100	Hy-AS-100	3	R
47	Mu-AS-47	23	MS	101	Hy-AS-101	3	R
48	Hy-AS-48	65	HS	102	Hy-AS-102	5	R
49	Hy-AS-49	35	S	103	Hy-AS-103	12	MR
50	Hy-AS-50	75	HS	104	Hy-AS-104	5	R
51	Hy-AS-51	82	HS	105	Hy-AS-105	7	MR
52	Hy-AS-52	43	S	106	Hy-AS-106	2	R
53	Hy-AS-53	32	S	107	Hy-AS-107	5	R
54	Hy-AS-54	30	S	108	Super Basmati	51	HS
				109	RICF-160	20	MS

Column with mean values D (%) at P ≤ 0.05. D%, Disease percentage; R, Resistant; MR, Moderately Resistant; MS, Moderately Susceptible; S, Susceptible; HS, Highly Susceptible.

### Biochemical parameters

Selected forty five mutants and hybrid genotypes (on the basis of IRRI standard disease rating scale) were tested for various biochemical parameters for identification of resistance level (resistance, moderate resistance, moderate susceptible, susceptible and highly susceptible).

#### Enzymatic antioxidants

**Fig 2:**
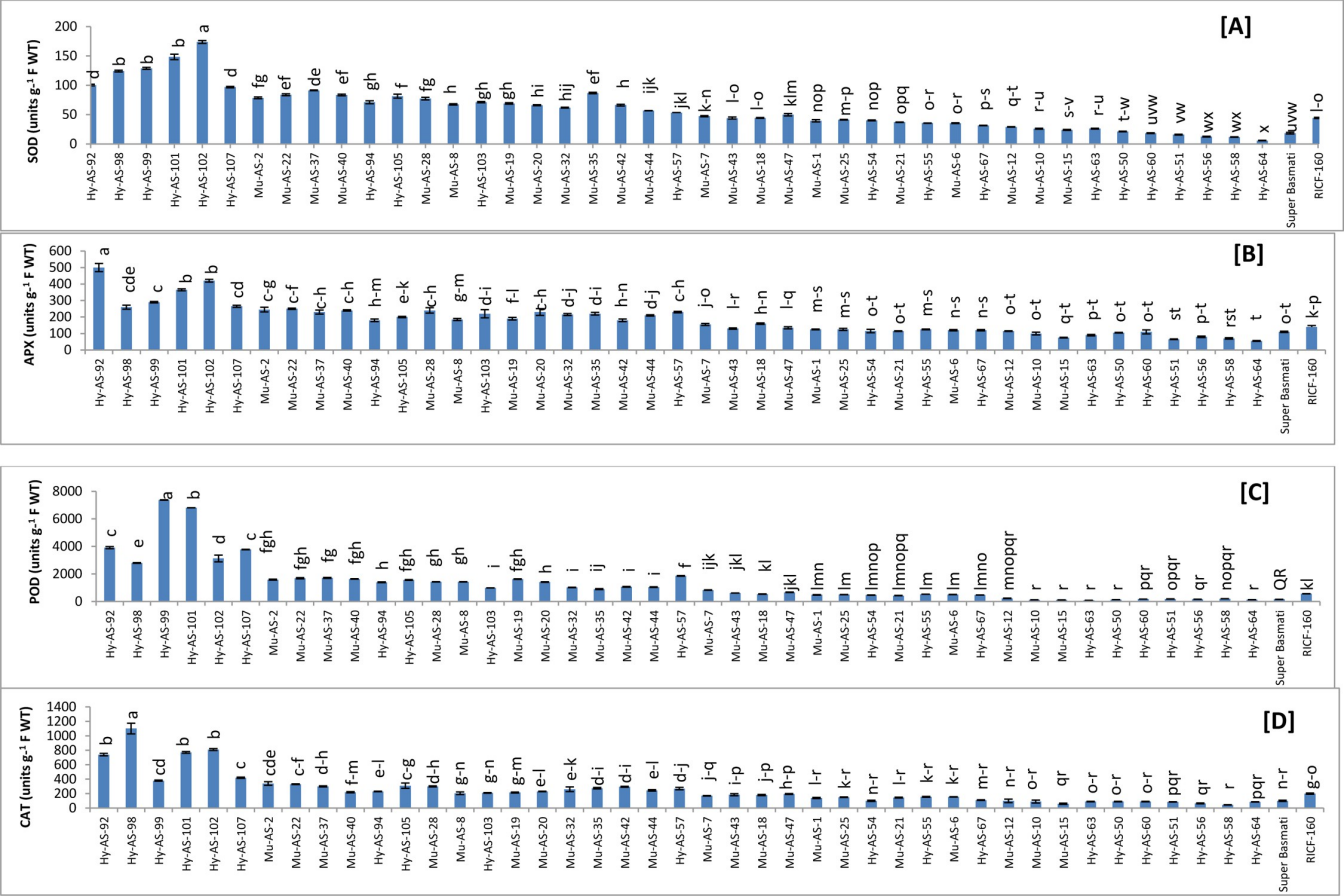
Comparison of superoxide dismutase (SOD) (A), ascorbate peroxidase (APX) (B), peroxidase (POD) (C) and catalase (CAT) (D) in rice genotypes (mean value ± SE), p<0.05).

*SOD*. The highest SOD activity was recorded in Hy-AS-102 (173.91±1.43 Units g^-1^ F WT) and Hy-AS-101 (148.44±1.44 Units g^-1^ F WT) followed by Hy-AS-99 (128.99±1.63 Units g^-1^ F WT) under the stress condition whereas in Super Basmati, SOD activity was observed as 18.5±1.43 (Units g^-1^ F WT). It was noted Hy-AS-64 (6±1.43 Units g^-1^ F WT) had the lowest SOD activity. Mu-AS-37 (91.50±0.61) and Mu-AS-35 (87±1.22) exhibited higher activity than the RICF-160 (44.50±1.02 Units g^-1^ F WT) **(Fig 2A).**

*APX*. APX activity was highest in Hy-AS-92 (500±24 Units g^-1^ F WT) and Hy-AS-102 (420±12 Units g-1 F WT) followed by Hy-AS-101 (365±4 Units g-1 F WT). However, in Super Basmati, APX activity was observed to be (110±2 Units g-1 F WT). The lowest APX activity was detected in Hy-AS-64 (55±8.2 Units g-1 F WT). It was recorded that APX activity was significantly lower in RICF-160 (140±8.2 Units/g f. wt.) than other twenty four genotypes see **Fig 2B**.

*POD*. Under the stress condition, maximum value of POD was found in Hy-AS-99 (7375±75.6 Units g-1 F WT) and Hy-AS-101 (6805.5±26.9 Units g-1 F WT) followed by Hy-AS-92 (3914.8±15 Units g-1 F WT) but in Super Basmati the POD value was recorded as 160±6.1 Units g-1 F WT The minimum POD activity was detected in Hy-AS-63 (85±10.2 Units g-1 F WT). However, these genotypes Mu-AS-20, Mu-AS-35, Hy-AS-92 and Hy-AS-102 showed moderate resistant to resistant phenotype to brown spot as well as had higher POD activity than RICF-160 (565±20 Units g-1 F WT) (**Fig 2C**).

*CAT*. Highest CAT activity was noted in Hy-AS-93 (1100±30 Units g^-1^ F WT) However, CAT activity in Super Basmati was observed as 100±20 Units g^-1^ F WT. The Hy-AS-58 (45±8.2 Units g^-1^ F WT) had the lowest CAT activity. Fifteen genotypes with CAT activity ranging from 340 to 205 (Units g^-1^ F WT) exhibited as higher range than RICF-160 (200±4.1 Units g^-1^ F WT) **(Fig 2D**).

The results exhibited that defense associated enzymatic antioxidants (SOD, APX, POD and CAT) were at maximum concentration in resistant phenotypes such as Hy-AS-102, Hy-AS-92, Hy-AS-99 and Hy-AS-93 followed by the moderately resistant (Mu-AS-2, Mu-AS-22, Mu-AS-35 and Mu-AS-37) and susceptible (Hy-AS-64 and Hy-AS-58) rice lines. However, enzyme accumulation varied between the five resistant genotypes. The susceptible and highly susceptible genotypes had a significantly lower range than RICF-160.

#### Non-enzymatic antioxidants

**Fig 3:**
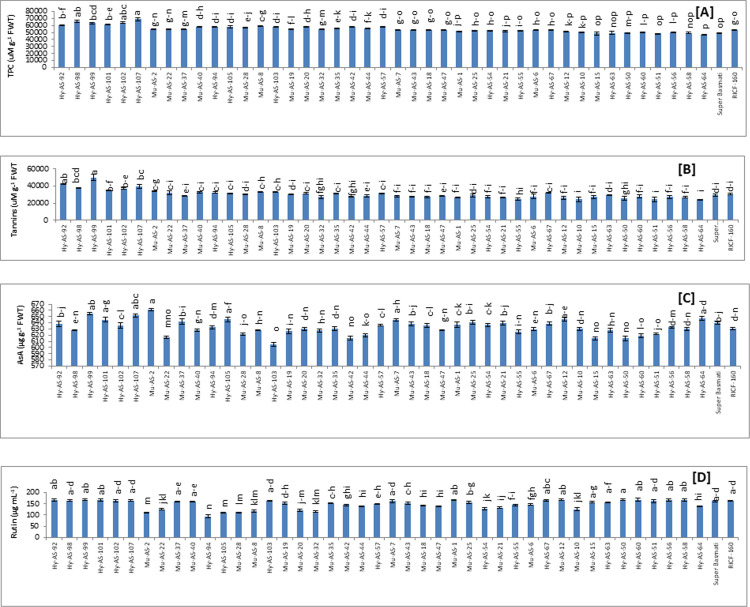
Comparison of total phenolic content (TPC) (A), tannins (B), ascorbic acid (ASA) (C) and total flavonoid content (Rutin) (D) in rice genotypes (mean value ± SE), p<0.05).

*TPC*. Among the forty five genotypes, six genotypes were grouped on the basis of resistant phenotypes range from 68800 to 60715 uMg^-1^ F WT. Mean values for TPC are presented in **Fig 3A**. Among these genotypes, the highest TPC was recorded in Hy-AS-107 (68800±210 uMg^-1^ F WT). Sixteen genotypes with TPC ranging from 58885 to 54835 (uMg^-1^ F WT) were exhibited moderate resistant phenotypes. Nineteen genotypes had the lowest TPC as compared to parent RICF-160. The lowest value was observed in Hy-AS-64 (46891±523 uMg^-1^ F WT).

*Tannins*. Among forty five genotypes, six genotypes fall in the resistant category in which the utmost value of tannins was found in Hy-AS-92 (42900 μuMg^-1^ F WT and sixteen in moderate resistant category in which Mu-AS-2 (33700 μuMg^-1^ F WT had the highest value whereas in Super Basmati was 2920 μuMg^-1^ F WT. The lowest tannins value was detected in Hy-AS-64 (24192 μuMg^-1^ F WT) (**Fig 3B**).

*AsA*. Considerably, the highest AsA content was in Mu-AS-2 (661 μgg^-1^ F WT) and Hy-AS-99 (654 μgg^-1^ F WT) followed by Hy-AS-107 (651.8 μgg^-1^ F WT). In Super Basmati, AsA content was observed as 639 μgg^-1^ F WT However, the lowest AsA content was noticed in Hy-AS-103 (605.3 μgg^-1^ F WT) that was significantly lower than RICF-160 (630 μgg^-1^ F WT) **(Fig 3C)**.

*TFC*. The Mu-AS-2 and Hy-AS-99 (168 μg/mL.) had the highest TFC as well as exhibited moderate resistant and resistant response to brown spot. The TFC in Super Basmati and RICF-160 were recorded as 161 and 162 μg/mL respectively. The Hy-AS-94 (93 μg/mL) had the lowest TFC **(Fig 3D**).

#### Hydrolytic enzymes

**Fig 4:**
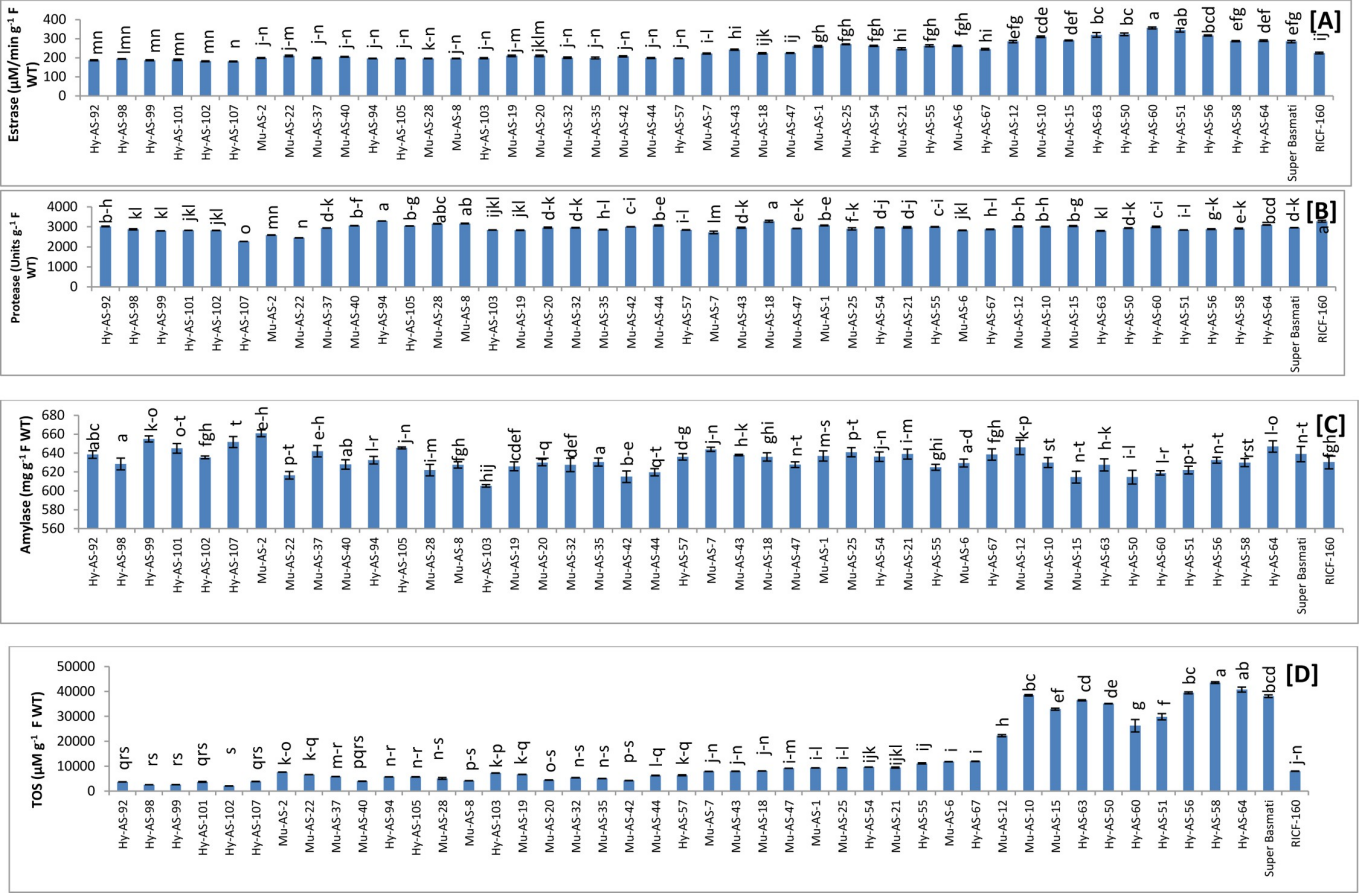
Comparison of esterase (A), protease activity (B), alpha amylase activity (C) and total oxidant status (TOS) (D) in rice genotypes (mean value ± SE), p<0.05).

*Esterase activity*. Esterase activity was determined among the forty five genotypes to reveal the effect of brown spot disease. Under the *B*. *oryzae* stress condition, twenty four genotypes had the lowest esterase activity than RICF-160 (225±2 μM/min g^-1^ F WT) in which the values range from 181 to 224 (μM/min g^-1^ F WT). Among these genotypes lowest esterase activity was in Hy-AS-107 (181±2.7 μM/min g^-1^ F WT). In Super Basmati, the esterase value was observed as (285±2.4 μM/min g^-1^ F WT). The highest esterase value was detected in Hy-AS-60 (357±4.1 μM/min g^-1^ F WT) **(Fig 4A)**.

*Protease activity*. Protease activity was estimated among the forty five genotypes to determine the effect of brown spot disease. Under the *B*. *oryzae* stress condition, Hy-AS-94 (3290±16 Units g^-1^ F WT) had maximum protease activity than RICF-160 (3280±8 Units g^-1^ F WT). In Super Basmati, the protease activity was observed as (2960±24 Units g^-1^ F WT). Hy-AS-107 (2270±49 Units g^-1^ F WT) had the minimum activity see **Fig 4B**.

*Alpha-amylase activity*. Under *B*. *oryzae* inoculum pressure, alpha amylase activity was recorded among forty five genotypes to reveal the effect of brown spot disease. Twenty three genotypes had a higher range of alpha-amylase than RICF-160 (630±0.75 mg g^-1^ F WT), in which Mu-AS-2 (661±3.96 mg g^-1^ F WT) had utmost activity while in Super Basmati; alpha-amylase activity was observed as (639±0.94 mg g^-1^ F WT). The lower range of alpha-amylase was in disease resistant genotype Hy-AS-103 (605±7.15 mg g^-1^ F WT) see **Fig 4C**.

Hydrolytic enzymes such as esterase, protease and alpha amylase play a crucial role in plant development and physiological processes. The results exhibited that protease and alpha amylase activity had higher in resistant and moderate resistant phenotypes to brown spot disease than Parent RICF-160 and Super Basmati.

#### Other biochemical parameters

*TOS*. TOS was determined among the forty five genotypes to reveal the effect of brown spot disease. Twenty four genotypes had the lowest TOS values than RICF-160 (8000±29 μM g^-1^ F WT) in which the values range from 2050 to 7600 (μM g^-1^ F WT). Among these genotypes lowest TOS activity was in Hy-AS-102 (2050±41 μM g^-1^ F WT). In Super Basmati, the TOS was observed as (38100±388 μM g^-1^ F WT). The highest TOS was detected in Hy-AS-58 (43550±82 μM g^-1^ F WT) see **Fig 4D**.

**Fig 5:**
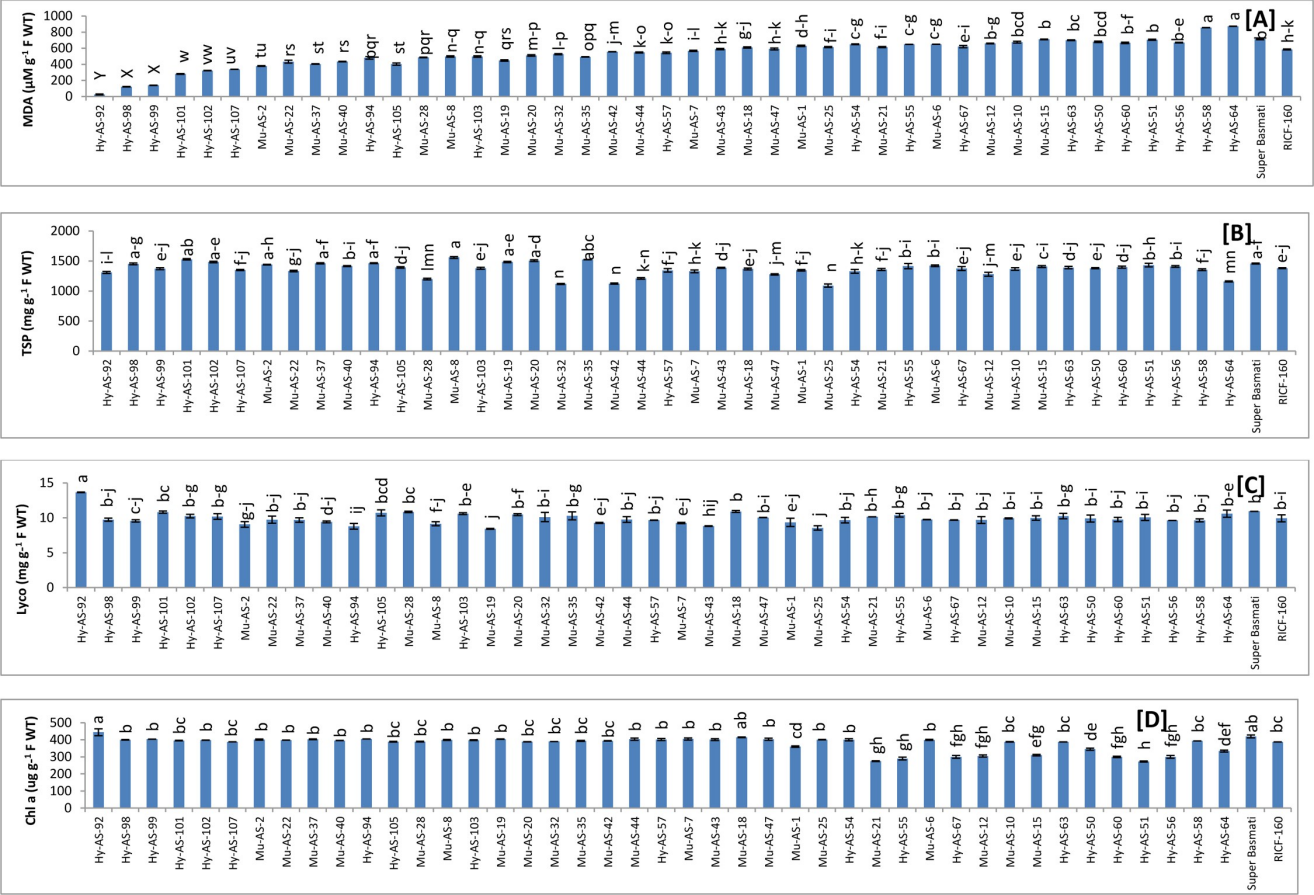
Comparison of MDA content (A), total soluble protein (TSP) (B), lycopene content (C) and chlorophyll ‘a’ (D) in rice genotypes (mean value ± SE), p<0.05).

*MDA*. MDA was observed among the forty five genotypes to depict the effect of brown spot disease. Twenty three genotypes had minimum MDA content than RICF-160 (585±8.2 μM g^-1^ F WT) in which the values range from 26 to 557 (μM g^-1^ F WT). Among these genotypes minimum MDA content was in Hy-AS-92. In Super Basmati, the MDA was observed as (712.6±1.3 μM g^-1^ F WT). The highest MDA was detected in Hy-AS-64 (873±6.1 μM g^-1^ F WT). Mean data presented in **Fig 5A**.

The results of TOS and MDA depicted the significant difference between the resistant and susceptible genotypes. It showed that resistant (Hy-AS-92, Hy-AS-102 and Hy-AS-107) and moderate resistant (Mu-AS-19, Mu-AS-20, Mu-AS-8 Mu-AS-35 and Mu-AS-37) genotypes had minimum content of TOS and MDA.

*TSP*. TSP was noted among forty five genotypes to depict the effect of brown spot disease. Twenty one genotypes had the higher content of TSP than RICF-160 (1381±22.7 mg g^-1^ F WT), in which Mu-AS-8 (1559±17) had maximum content. TSP content was observed 1458±9.9 (mg g^-1^ F WT) in Super Basmati. The minimum content of TSP was in Mu-AS-25 (1091±7.5 mg g^-1^ F WT) see **Fig 5B**.

#### Pigment analysis

*Lycopene content*. Lycopene content was determined among the forty five genotypes to reveal the effect of brown spot disease. Lycopene content was observed to be non-significant in all the genotypes under the *B*. *oryzae* stress condition. Nineteen genotypes had maximum content than RICF-160 (9.93±0.408 mg g^-1^ F WT) in which the values range from 13.66 to 9.98 (mg g^-1^ F WT). Among these genotypes maximum lycopene content was in Hy-AS-92 (13.66±0.43 mg g^-1^ F WT). In Super Basmati was observed as 10.93±0.222 mg g^-1^ F WT. The minimum content was detected in Mu-AS-19 (357±4.1 mg g^-1^ F WT) **(Fig 5C)**.

**Fig 6:**
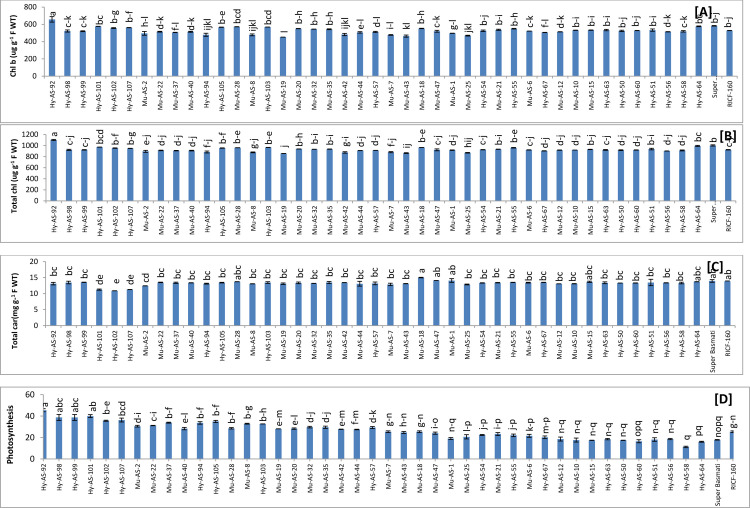
Comparison of chlorophyll b (A), total chlorophyll (B), total carotenoids (C) and photosynthesis rate (D) in rice genotypes (mean value ± SE), p<0.05.

*Chlorophyll a and Chlorophyll b*. Chlorophyll ‘a’ was determined among the forty five genotypes to reveal the effect of brown spot disease. Chlorophyll “a” was not significantly different among all the genotypes (45) under fungal stress conditions **(Fig 5D)**. Fig In Chlorophyll ‘b’ Nineteen genotypes had maximum content than RICF-160 (528±8.3 ug g^-1^ F WT) in which the values range from 658 to 532 (ug g^-1^ F WT). Among these genotypes maximum chlorophyll ‘b’ content was in Hy-AS-92 (658±31.6 ug g^-1^ F WT). In Super Basmati was observed as 583±12.1 ug g^-1^ F WT. The minimum content was detected in Mu-AS-19 (452±1.2 ug g^-1^ F WT) **(Fig 6A)**.

In the case of total chlorophyll, the same trend was observed and means data was presented in **Fig 6B**.

*Total carotenoid*. Total carotenoid was determined among the forty five genotypes to depict the effect of brown spot disease. Under the *B*. *oryzae* stress condition, four genotypes had maximum value than RICF-160 (13.8±0.009 mg g^-1^ F WT) in which the values range from 15 to 14 (mg g^-1^ F WT). Among these genotypes, maximum value was in Mu-AS-18 (15±0.389 mg g^-1^ F WT). In Super Basmati, the carotenoid value was 285±2.4 mg g^-1^ F WT. The minimum content was seen in Hy-AS-102 (10.9±0.110 mg g^-1^ F WT). Total carotenoid content was not significantly different from one another in forty five genotypes (**Fig 6C**).

*Photosynthesis rate*. Photosynthetic rate was measured in the forty five genotypes along with Super Basmati and RICF-160 to reveal the effect of brown spot disease. Genotypes condition revealed that Hy-AS-102 had the maximum photosynthetic rate (22.23±0.49 U mol m^-2^ S^-1^), followed by Hy-AS-92 (45.5±1.4 U mol m^-2^ S^-1^) and Hy-AS-101 (40±1.3 U mol m^-2^ S^-1^). RICF-160 and Super Basmati’s photosynthetic rate were found to be 25.4±0.8 and 17.8±0.3 (U mol m^-2^ S^-1^) respectively. Hy-AS-58 (11.2±1.4 U mol m^-2^ S^-1^) had the lowest rate of photosynthesis **(Fig 6D)**.

### Correlation analysis (Pearson test)

**Fig 7:**
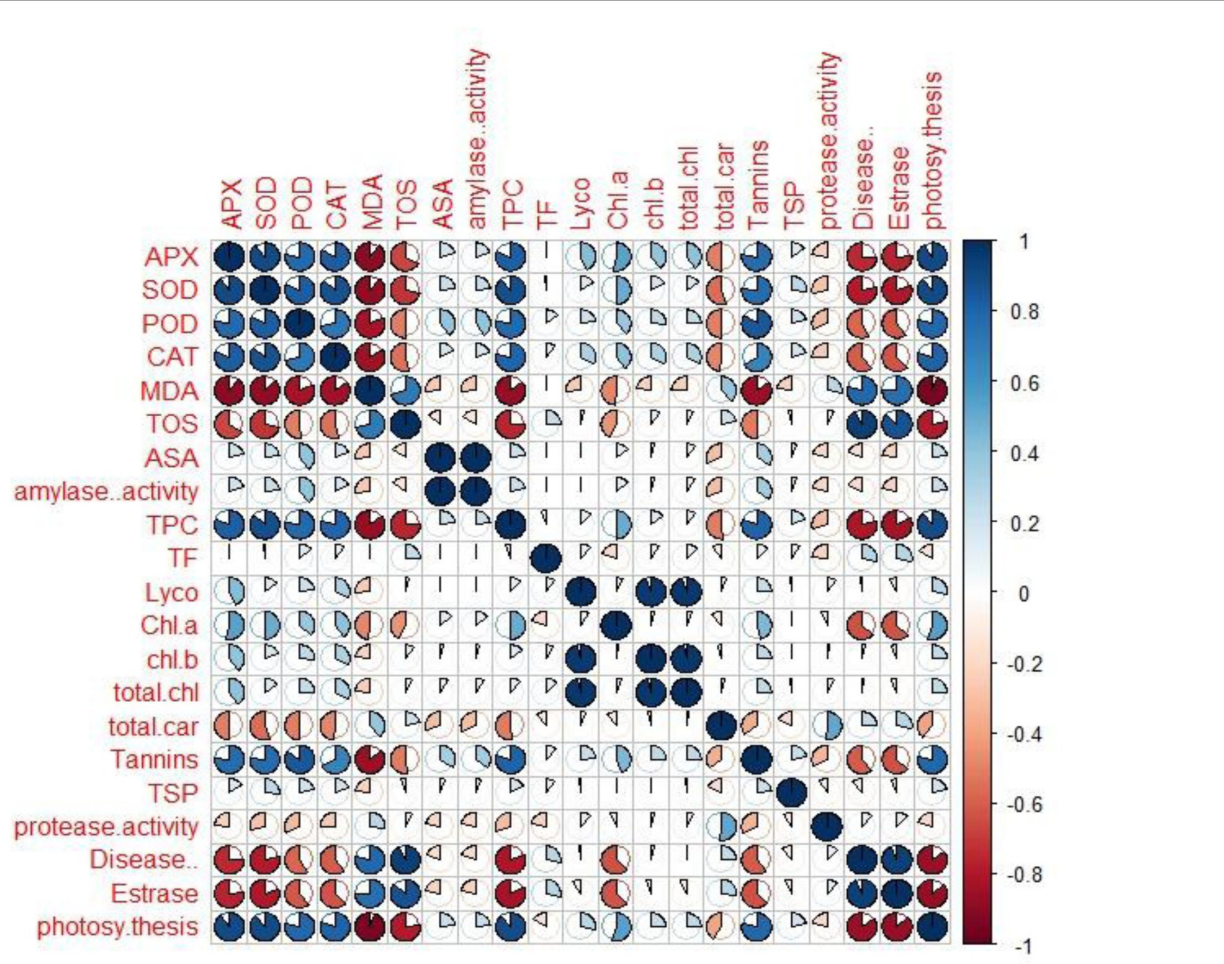
Pearson Correlation matrix showing Pearson’s correlation among physiological and biochemical traits in rice genotypes under fungal stress condition. Red and blue circles represent the negative and positive correlation, respectively. The extent of correlation is directed by pie filled area, i.e., smaller to larger pie fill area represents low to high correlation. APX (ascorbate peroxidase), SOD (superoxide dismutase), POD (peroxidase), CAT (catalase), MDA (malondialdehyde), TOS (total oxidant status), AsA (ascorbic acid), TPC (total phenolic content), TF (total flavonoids), Lyco (lycopene content), chl a (chlorophyll a), chl b (chlorophyll b), total chl (total chlorophyll), total car (total carotenoids) and TSP (total soluble protein).

Correlation (Pearson test) for physiological and biochemical traits under biotic (*B*. *oryzae*) stress condition was performed by using R studio with 95% confidence interval **(Fig 7)**.

Among forty five genotypes under fungal stress condition, positive correlation with physiological and biochemical traits was expressed in bold form and negative correlation in bold form with negative signatures. So that the significant correlation among genotypes under biotic stress with physiological and biochemical traits related to stress resistance was easily identified (Table 3).

**Table 3:** Correlation matrix for physiological and biochemical traits.

	APX	SOD	POD	CAT	MDA	TOS	ASA	amylase activity	TPC	TF	Lyco	Chl a	chl b	total chl	total car	Tannins	TSP	protease activity	Disease %	Esterase
SOD	**0.90**																			
POD	**0.78**	**0.83**																		
CAT	**0.83**	**0.86**	**0.72**																	
MDA	**-0.90**	**-0.89**	**-0.82**	**-0.85**																
TOS	**-0.67**	**-0.71**	**-0.51**	**-0.54**	**0.71**															
ASA	0.20	0.23	**0.39**	0.18	-0.26	-0.15														
Amylase activity	0.20	0.23	**0.39**	0.19	-0.27	-0.15	1.00													
TPC	**0.81**	**0.88**	**0.77**	**0.79**	**-0.86**	**-0.75**	0.22	0.22												
TF	0.01	-0.02	0.14	0.10	-0.01	0.25	0.00	0.00	-0.05											
Lyco	**0.43**	0.16	0.22	0.31	-0.26	0.04	0.00	0.00	0.12	0.10										
Chl a	**0.54**	0.50	**0.38**	0.40	-0.48	-0.44	0.14	0.14	0.49	-0.19	0.08									
chl b	**0.39**	0.19	0.25	0.32	-0.22	0.10	0.04	0.04	0.14	0.09	**0.95**	0.03								
total chl	**0.41**	0.16	0.25	0.32	-0.24	0.07	0.09	0.09	0.11	0.13	**0.96**	0.06	0.97							
total car	**-0.50**	**-0.55**	**-0.50**	**-0.48**	0.38	0.22	-0.31	-0.31	**-0.51**	-0.10	0.06	-0.11	-0.04	0.03						
Tannins	**0.77**	**0.76**	**0.84**	**0.67**	**-0.86**	**-0.52**	0.34	0.34	**0.80**	0.13	0.24	**0.45**	0.25	0.26	-0.36					
TSP	0.16	0.27	0.21	0.19	-0.23	-0.04	0.05	0.06	0.18	0.10	-0.02	0.00	0.00	-0.02	-0.18	0.22				
protease activity	-0.23	-0.28	-0.32	-0.26	0.28	0.08	-0.21	-0.21	-0.30	-0.23	0.10	-0.06	0.03	0.09	**0.51**	-0.33	-0.09			
Disease %	**-0.75**	**-0.79**	**-0.58**	**-0.61**	**0.79**	**0.94**	-0.19	-0.19	**-0.80**	0.28	-0.02	**-0.63**	0.03	0.01	0.25	**-0.61**	-0.10	0.12		
Estrase	**-0.77**	**-0.80**	**-0.61**	**-0.62**	**0.75**	**0.86**	-0.22	-0.22	**-0.82**	0.28	-0.09	**-0.62**	-0.06	-0.07	0.28	**-0.63**	-0.07	0.12	**0.93**	
photosy thesis	**0.90**	**0.89**	**0.79**	**0.80**	**-0.94**	**-0.78**	0.23	0.23	**0.89**	-0.15	0.28	**0.55**	0.25	0.25	**-0.40**	**0.79**	0.21	-0.20	**-0.86**	**-0.86**

Values in bold are different from 0 with a significance level alpha = 0.05

Under biotic stress condition, SOD, POD, CAT and TPC were positively correlated with APX and were negatively with MDA and TOS, indicating that high activity of antioxidant enzymes like SOD, POD, CAT and APX reduced the oxidative damage in the form of lipid peroxidation. MDA (an indicator of oxidative damage) was positively correlated with TOS. This correlation directly showed that with the increase in MDA, a relative amount of TOS also increased and ROS in disease susceptible genotypes was increased under stress condition.

Positive correlation was recorded between POD with AsA and alpha amylase and between total carotenoids and protease. Positive correlation was recorded between lycopene with chl a, chl b and total chlorophyll. Total carotenoids were negatively correlated with APX, SOD, POD, CAT and TPC. Photosynthesis positively correlated to SOD, POD, APX, CAT, TPC, chl ‘a’ and tannins and negatively with MDA, TOS, disease damage (%) and esterase activity.

The positive correlation was observed tannins with APX, SOD, POD, CAT, TPC and chl ‘a’ and negatively with MDA and TOS. Disease damage (%) and esterase activity were positively correlated with MDA and TOS, and negatively with APX, SOD, POD, CAT, TPC, chl ‘a’ and tannins. The antioxidants scavenge reactive oxygen species generated in plant under stress condition, so positive association between SOD, CAT, TPC, POD and APX activity decrease the disease damage (%) in resistant genotypes. These antioxidants will help to combat the ROS level in the resistant genotypes. These attributes could serve as stress markers. Early detection of disease resistance in the breeding material on the basis of identified antioxidant markers. It helps the breeder to squeeze the population for evaluation in the succeeding generations.

### Cluster analysis

**Fig 8:**
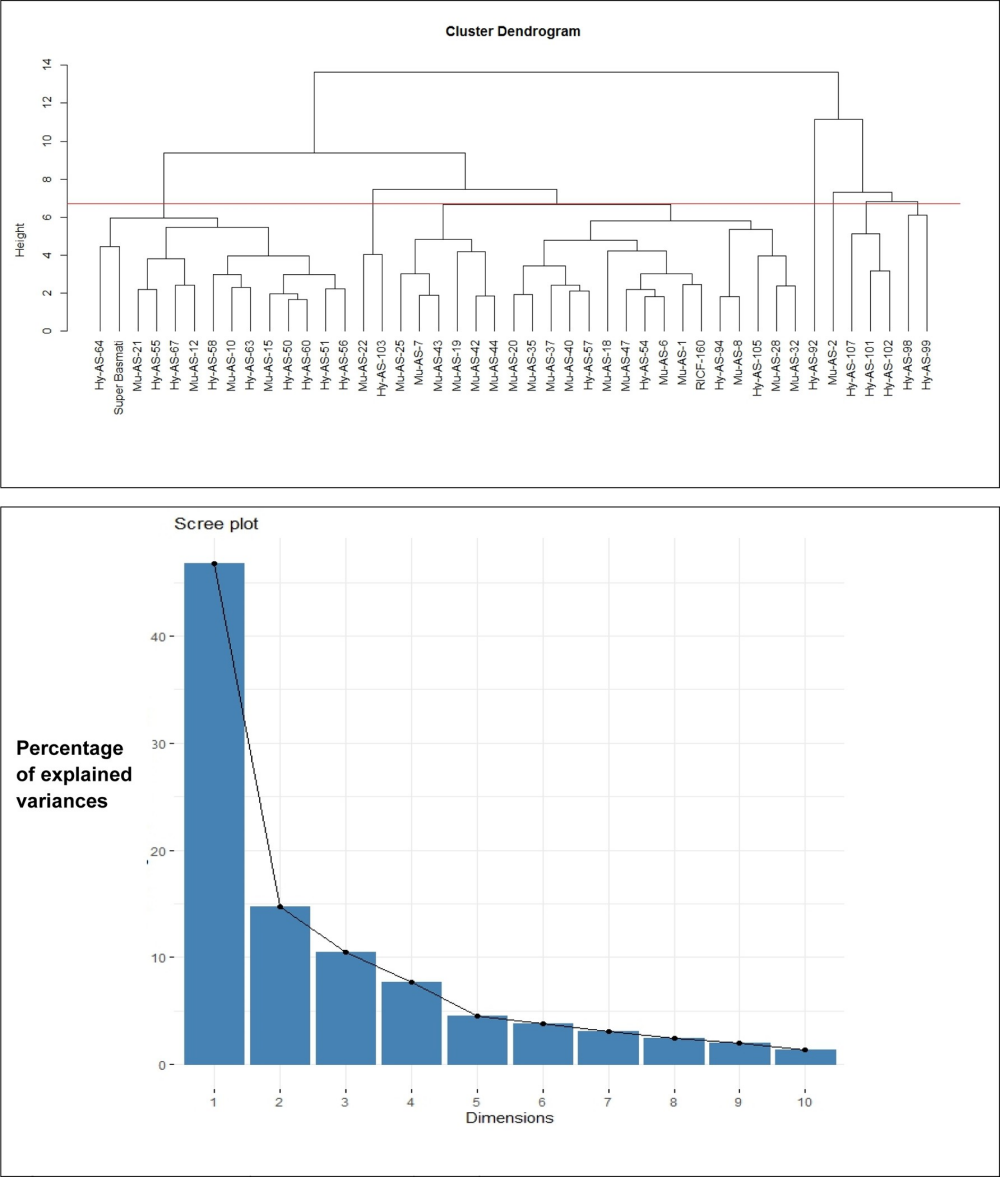
**A:** Tree diagram based on physiological and biochemical traits for different rice genotypes under biotic stress condition. **B:** Scree plot representing cumulative variability and Eigenvalues for studied parameters.

Clustering of rice genotypes was performed on the basis of physiological and biochemical traits under biotic stress condition (Fig 8A). Cluster analysis grouped forty five genotypes into four clusters. Fourteen genotypes in Cluster I followed by two, twenty two and seven genotypes, respectively in clusters II, III and IV.

### Principal component biplot analysis

To reduce the dimensionality of the data, the raw data were transformed into major factors in order to assess genetic variability and learn more about the relationships between the variables. All parameters under consideration were used in a principal component analysis (PCA). Scree plot **(Fig 8B)** revealed that four PC-I, PC-II, PC-III and PC-IV of the 21 major components had extracted Eigenvalues >1. The number of components we need to extract can be determined using eigenvalues, which are a measure of the variance that contributes for each factor. Since the Eigenvalues of the remaining principal components were less than 1, they were not further addressed. These four main components collectively accounted for 79.693% of the genetic resources total variation. The major contributions to a cumulative variability of 100% were PC-I and PC-II, each of which had a value of 61.526%. The most crucial component, PC-I (46.77%), was responsible for explaining the most variation **(Table 4)**.

**Table 4:** Principal component analysis for physiological and biochemical traits in different rice genotypes.

	Dim.1	Dim.2	Dim.3	Dim.4	Dim.5	Dim.6	Dim.7	Dim.8	Dim.9	Dim.10	Dim.11	Dim.12	Dim.13	Dim.14	Dim.15	Dim.16	Dim.17	Dim.18	Dim.19	Dim.20	Dim.21
**Eigenvalue**	9.822	3.099	2.208	1.607	0.952	0.801	0.642	0.52	0.412	0.282	0.174	0.152	0.093	0.081	0.046	0.038	0.029	0.025	0.011	0.007	0
**Variability (%)**	46.771	14.755	10.516	7.651	4.532	3.813	3.055	2.478	1.961	1.343	0.83	0.724	0.441	0.385	0.221	0.18	0.14	0.118	0.053	0.032	0.001
**Cumulative %**	46.771	61.526	72.042	79.693	84.225	88.037	91.093	93.571	95.532	96.874	97.705	98.429	98.87	99.255	99.475	99.655	99.795	99.914	99.967	99.999	100

**Fig 9:**
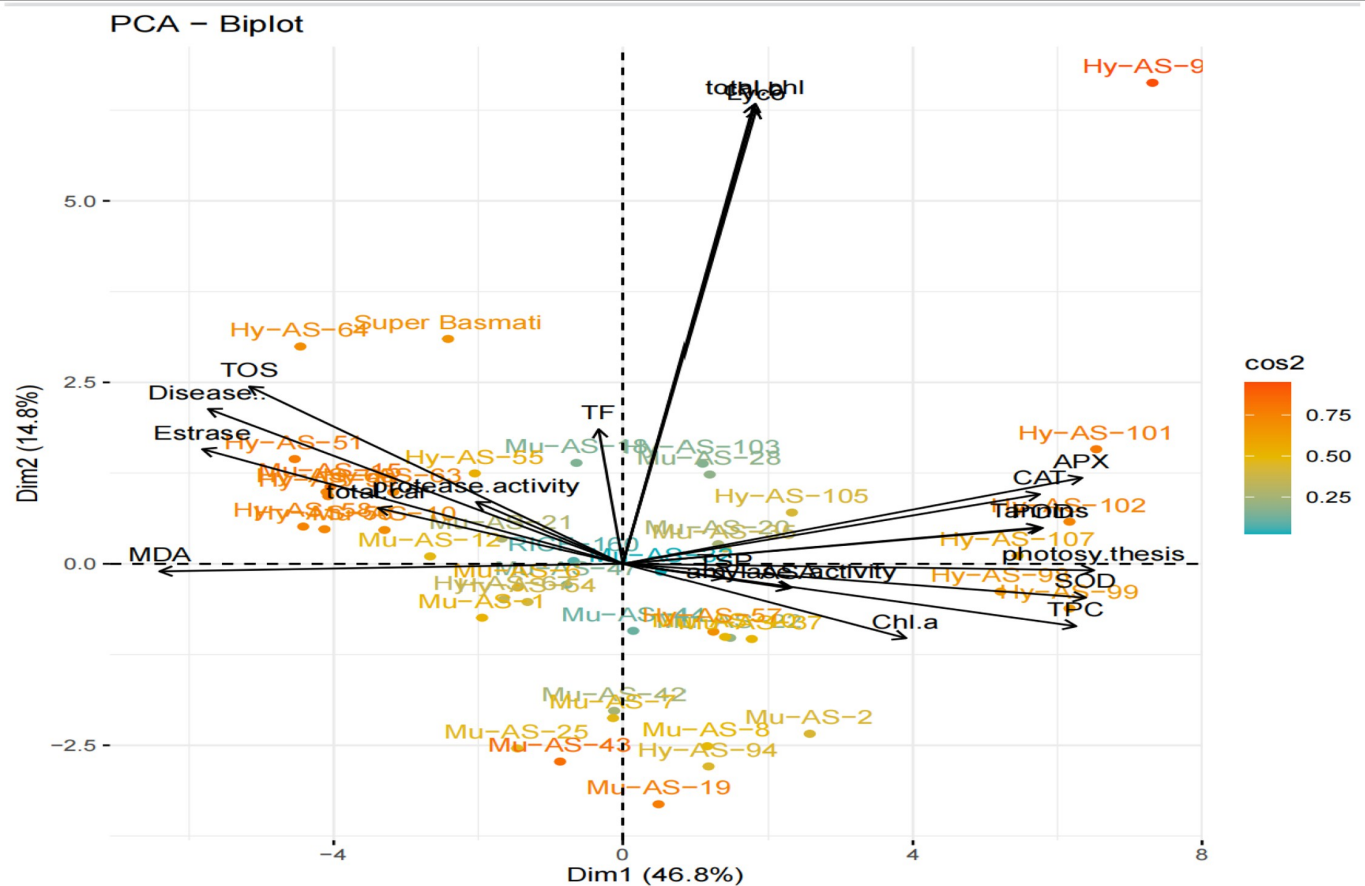
Bi-plot illustration of rice genotypes for the first two principal components.

The F1 scores (x-axis) and F2 scores (y-axis) for each trait were plotted in a biplot on genotype keeping in mind the characteristic. The visual comparison of all genotypes based on multiple traits was efficiently demonstrated by this genotype by trait biplot, which also demonstrated the correlations between the traits. Important information was extracted using the angles between the vectors and the distance of the genotypes from the biplot’s origin. When the angle between two trait vectors is less than 90°, there is a positive correlation between the traits; when the angle is more than 90°, there is a negative correlation; and when the angle is equal to 90°, and then traits show no dependency on each other **(Fig 9)**. The F1 was positive loaded for APX, SOD, POD, CAT, AsA, amylase, TPC and TF. Moreover, F1 and F2 responsible for 46.8% and 14.8% respectively variation among genotypes in which enzymatic antioxidants in F1 and TF activities in F2 have a major contribution.

## Discussion

The productivity of crops was dramatically impacted globally by an increase in different environmental challenges, predominantly the abiotic and biotic stresses [37]. The development of new rice varieties with resistance to water stress and diseases particularly the brown spot in near future are in dire need for sustainable production [38–40]. The main objectives of this study were to screen the rice mutants and hybridized material using biochemical profiling as a marker under brown spot inoculum pressure at the seedling stage.

Reactive oxygen species (ROS) are produced excessively in living organisms’ cells, tissues and extracellular matrix, which sets off a series of events that strengthen the body’s natural defense (such as enzymatic and non-enzymatic antioxidants) and successfully neutralize toxic ROS [41, 42]. The detoxification of hydrogen peroxide is carried out by the enzyme ascorbate peroxidase (APX), which is a crucial part of the glutathione ascorbate cycle [43]. It helps in the protection of plant cells from environmental stress [44, 45]. As in our study low APX activity was detected in disease susceptible genotypes such as Hy-AS-64 and Hy-AS-51 while resistant (a total of six genotypes) and moderate resistant (sixteen) genotypes have higher APX activity as compared to Super Basmati and RICF-160 to combat the ROS. Similar findings were noted in the leaves of rice genotypes that cause an increase of total APX activities under stress. It appeared due to preferential induction of APX-4 to APX -7 isoforms [46]. The boost up of APX activity and expression by oxidative stress have also been observed in the germinating rice embryos [47].

All living things, especially higher plants, have CAT activity. Due to oxidative stress, CAT is found in key locations such as mitochondria, peroxisomes, chloroplasts, and cytosol where it breaks H_2_O_2_ into oxygen and water [48]. Lowest CAT activity was found in highly disease susceptible genotype Hy-AS-58 while high CAT activity in fifteen genotypes as compared to RICF-160 and thirty three genotypes to Super Basmati. In general, the highest CAT activity (1100± 16.3 Units g^-1^ F WT) was found in a Hy-AS-93 which is resistant to brown spot disease. Similar results were also revealed in mutant rice genotype HTT-121 under heat stress in which increased CAT activity was perceived [49]. It is possible that increased proteolysis, which is induced during the infection process, is the cause of the decrease in CAT activity in susceptible rice genotypes [50]. The role of CAT in the plant-pathogen interaction appears to be more complex. However, the difference in the CAT activity between rice genotypes suggests that this enzyme plays a major role in the rice resistance to brown spot disease. This result is in line with [51], They showed that the CAT activity was higher in the leaves of *Aspergillus flavus*-resistant maize lines than in susceptible ones. The comparison of biotic to abiotic stress, which involves a correlation between CAT activity and plant resistance [52].

Peroxidases (POD) are enzymes that use free radicals to conduct an oxidation-reduction reaction, converting various chemicals into the polymerized or oxidized state [53, 54]. Due to their vital role in physiological processes like the regulation of growth by lignification, the cross-linking of pectin and structural proteins in cell walls, and the catabolism of auxins, plant peroxidases have been used as biochemical markers for a variety of abiotic as well as biotic stresses [55, 56]. The findings of our investigation demonstrated that resistant (Hy-AS-92, Hy-AS-102, Hy-AS-98, Hy-AS-99, Hy-AS-101 and Hy-AS-107) and moderately resistant (a total of sixteen) rice genotypes have higher concentrations of the peroxidase enzyme. Lowest POD activity was found in is highly susceptible (Hy-AS-63) to brown spot disease, while highest POD activity in Hy-AS-99 and Hy-AS-101 was observed that resistance to brown spot disease. Similarly, in *Oryzae sativa* and green gram raised POD activity under heat stress and elevated temperature was examined [49, 57, 58]. The findings imply that the POD was crucial in the defense against brown spot disease. POD activity was induced in plant tissues after infection with plant pathogens, and resistant plants showed a greater rise than susceptible ones [59].

The antioxidant enzyme SOD work to prevent reactive oxygen species from oxidizing cellular components [60]. In our study, Lowest SOD activity was found in Hy-AS-64 which is also highly susceptible to brown spot disease. In general, the highest SOD activity, in Hy-AS-102 exhibited resistance phenotype to brown spot disease. In our studies, SOD activity increased in mutant and hybridized rice genotypes to combat the stress conditions. The increased production of reactive oxygen species could be a protective measure adopted by rice plants against oxidative damage. SODs showed increased activity in rice seedlings under mild as well as high drought stress. Earlier [61], have comparable findings that SOD activity increased under many stressful conditions. Similar results were found for increased SOD activity in rice under stress through foliar application of proline [21, 62, 63].

TPC and TFC are the two most important bioactive molecules among non-enzymatic antioxidants. These compounds play different structural roles and are closely linked to antioxidant activity. They may be found in all parts of plant [64, 65]. In the phenolic fraction of brown rice, phenolic acids play a significant role. These include hydroxybenzoic and hydroxycinnamic acids, both of which may have health benefits [66, 67]. In general, the highest TPC was found among hybrids and mutants are Hy-AS-107 and Mu-AS-8. These genotypes showed the resistance to moderate resistant phenotype against brown spot disease. The outcomes indicate antioxidant activities, flavonoids and total phenols shown a remarkable connection with the disease resistance strength of rice. Their increases in fungal stress were found proportional to fungal resistance levels [68, 69]. Earlier, [70] observed that black and red-hulled rice had radical-scavenging ability depended on the concentrations of anthocyanins and proanthocyanidins. The high levels of flavonoids (flavonols, quercetin and kaempferol) contents were correlated with enhanced stress tolerance ability of white clover under drought and UV-B radiation. An increase in TPC in variety Bhakhar-2011 in well-watered and low water stress conditions was noted by the earlier workers [71], that is consistent with the current study results. Previous studies highlighted that phenolic contents as antioxidants and sunshield plays a major role against water stress and ultraviolet radiation. The significant increase of the phenolic acids detected in our genotypes proposed that these phenolic contents were involved in the response of rice against fungal diseases.

Vitamin C, functions as a non-enzymatic antioxidant and facilitates the movement of electrons by scavenging ROS and regenerating the antioxidant form of vitamin E [72, 73]. One of the effective antioxidants against various stressors in plants, particularly in *Oryza sativa*, is AsA [74, 75]. In the study, low content of AsA was detected in Hy-AS-103 genotype which is resistant to brown spot disease. Eleven and twenty three genotypes exhibited high ascorbic acid as compared to Super Basmati and RICF-160 respectively. The highest ascorbic acid was found in Mu-AS-2, Hy-AS-99 and Hy-AS-107 that exhibited resistance response against brown spot disease. AsA was previously supposed to be quite rare in rice, but during the germination process, AsA rapidly increases up to 98.5 g/1g due to the reactivation of the AsA production in seeds. Its composition in rice plants can be changed by modifying the biosynthetic or recycling pathways for AsA. In addition AsA provide resistance to environmental stresses and is essential for tiller formation [76].

Hydrolytic enzymes, such as esterase, protease, and alpha-amylase, selectively break down big molecules into smaller ones within living organisms [77]. They can also act as a secondary system of antioxidants by repairing DNA and using damaged molecules [78]. The production and hydrolysis of ester bonds from a variety of substrates can be catalyzed by esterases, which are abundant and present in a wide range of organisms [79–81]. In current study, esterase exhibited minimum activity in resistant and moderate resistant phenotypes as compared to susceptible genotypes to disease.

Proteases play a crucial role in the entire process of protein turn over at every stage of a plant’s life cycle. Plant development and physiology depend on proteolytic enzymes, which also serve as a source of the amino acids needed to make new proteins [82]. Low protease activity was in Hy-AS-107 and highest protease activity was noted in Hy-AS-94, both of these genotypes had resistant and moderately resistant response to brown spot disease. It was previously documented that there was no direct link between increased proteolytic activity and disease susceptibility. The proteolytic response of these tolerant mutants was similar to that of sensitive strains. The increase in protease activity is probably not directly related to disease susceptibility or resistance. It may help with amino acid remobilization during grain filling. The methods utilized in this work to quantify proteolytic activity primarily identify proteins with vacuolar origin, whereas the chloroplastic cell compartment has the most protein. It’s possible that during grain filling, chloroplasts serve as the primary site for amino acid remobilization [83].

In order to succumb products like maltose and glucose, the hydrolase enzyme alpha-amylase catalyzes the hydrolysis of 4-glycosidic bonds with internal α-1 in starch. It can be separated from animals, plants, or microorganisms. Low alpha-amylase activity was found in Hy-AS-103, while highest alpha-amylase activity in Mu-AS-2. Our advanced rice mutant/hybrid genotypes have an excellent activity of alpha amylase. Similarly, earlier researchers [84] documented that physiological and anatomical alterations in mutant rice enhanced water stress resistance. Frequently, TOS is used to calculate overall level of oxidation [85, 86]. It is noted that genotypes had resistant and moderate resistant response to brown spot disease exhibited low TOS activity as compared to Super Basmati and Parent RICF-160. The highest TOS activity was observed in Hy-AS-58 and Hy-AS-64 and Hy-AS-56 were highly susceptible to brown spot disease. Our results are in line with the previous investigation on chickpea highlighted that under heat and low moisture stress exposure, the oxidant status of sensitive genotypes increased due to oxidative stress [87]. The mutants and hybrids used in this study have the survival ability under stress condition which indicates that the use of mutagens create variability in the genotypes.

Usually, MDA is used to identify membrane lipid peroxidation in plants which can damage cell wall integrity under stress condition [88, 89]. Low MDA content was found in twenty three and forty two genotypes as compared RICF-160 and Super Basmati, respectively. Resistant line (Hy-AS-92) had showed the lowest MDA content, while highest MDA content found in highly susceptible genotypes (Hy-AS-64) to disease [90, 91]. The results were in accord with an earlier report [92], in which chickpea heat tolerant genotypes had a lower accumulation of MDA as compared to sensitive genotypes.

Under stress, Stomatal closure that led to decrease in photosynthetic rate was reported. Stomatal closure decreased CO_2_ uptake and restricted rubisco activity, which in turn decreased photosynthesis [93]. Stress affected the plant growth and rice yield in this way. In our study, photosynthesis was significantly lower in Hy-AS-58, Hy-AS-64 and Mu-AS-25 but higher in Hy-AS-92, Hy-AS-101, Mu-AS-8 and Mu-AS-37. According to photosynthetic rate, Hy-AS-58, Hy-AS-64 and Mu-AS-25 were found to be more susceptible genotypes under stress condition. However, the resistant rice genotypes have more photosynthesis rate under fungal stress than susceptible genotypes.

Carotenoids exert an important role in protecting the photosynthetic part from photo-oxidation and represent essential constituents of the light harvesting and reaction center complexes in plants. In addition, Carotenoids give flowers and fruits their color, greatly enhancing plant-animal communication [94]. Low carotenoids content was found in Hy-AS-102, while highest carotenoids content found in Mu-AS-18 and Mu-AS-47. The earlier researchers reported that mutant rice genotypes "DD14" responded to water deficiency stress better than other cultivars in terms of chlorophyll a, total chlorophyll, total carotenoids, and fertile grain characteristics [95]. Low lycopene content was found in Mu-AS-19 followed by Mu-AS-25, while highest lycopene content found in Hy-AS-92 as compared to Super Basmati. Previously, Black, red, and white grains of rice had dramatically different flavonol, carotenoid, and anthocyanin concentrations. Depending on the color of the grain, the carotenoid content showed clear variances. Quality of the rice grain is now the top priority for both breeding programs and rice stakeholders. One method to enhance the nutritional value of rice grains is bio-fortification through conventional breeding. The variants with the highest flavonoid and carotenoid levels would be the most promising for future breeding purposes. With regard to grain color, flavonoid and carotenoid concentrations noticeably differ [96].

The activity of other non-enzymatic antioxidants such as lycopene, AsA, tannins, TOS, and TPC was significantly increased under fungal stress condition in resistant genotypes, i.e., Mu-AS-8, Mu-AS-20, Mu-AS-35, Hy-AS-92 and Hy-AS-98, Hy-AS-99, Hy-AS-101, Hy-AS-102, Hy-AS-107. The lesser accumulation of MDA content and higher activity of enzymatic antioxidants such as SOD, POD, and APX under stress-treated plants of Mu-AS-20, Mu-AS-35, Hy-AS-92 and Hy-AS-102 indicated that these genotypes had adaption capacity for brown spot disease under *B*. *oryzae* stress condition in comparison with susceptible genotypes, i.e., Mu-AS-15, Mu-AS-47, Hy-AS-50, Hy-AS-55, Hy-AS-58 and Hy-AS-64. Distance between genotype and bi-plot origin measures genotypic differences from the grand mean. The genotypes with long and short distance from the origin can be used to determine best or poorest performers in the variable climate conditions. Results revealed that genotypes Mu-AS-8, Mu-AS-20, Mu-AS-19, Mu-AS-35, Hy-AS-92, Hy-AS-98, Hy-AS-101 and Hy-AS-102 had long distance from the origin, viewing well performing genotypes with the reference to several traits under study. The genotypes found adjacent the origin of the bi-plot were poorer performers than the distant genotypes.

## Conclusion

The study revealed that under *B*. *oryzae* stress, the physiological and biochemical analysis confirmed the resistance of rice hybrids and mutants against brown spot disease. Positive correlations were observed among stress bio-markers and disease response. Rice genotypes i.e. Mu-AS-8, Mu-AS-19, Mu-AS-20 and Mu-AS-35 exhibited moderate resistant response while Hy-AS-92, Hy-AS-98, Hy-AS-99, Hy-AS-101, Hy-AS-102 and Hy-AS-107 showed resistant response to brown spot disease. Brown spot resistant rice genotypes with better productivity had lesser values of malondialdehyde and total oxidant status and higher antioxidant activities i.e. superoxide dismutase, peroxidase, total phenolic content and lycopene. The selected resistant genotypes had adaption and resistance capacity against *Bipolaris oryzae* stress because of better defense system resulting in higher productivity even under stressed conditions. In conclusion, identified resistant mutants i.e. Mu-AS-8, Mu-AS-19, Mu-AS-20 and Mu-AS-35 and hybrids i.e. Hy-AS-92, Hy-AS-98, Hy-AS-99, Hy-AS-101, Hy-AS-102 and Hy-AS-107 could be used in rice breeding program to achieve sustainable rice production by coping the emerging challenge of brown spot disease under variable climate conditions.

## Supporting information

S1 Table List of genotypes used in the study. (DOCX)

S2 Table List of genotypes used in biochemical study. (DOCX)
