# Supplementary material for: Identification of biochemical indices for brown spot (Bipolaris oryzae) disease resistance in rice mutants and hybrids
Source: PLoS One. 2024 Apr 18;19(4):e0300760. doi: 10.1371/journal.pone.0300760 (PMC11025958; doi:10.1371/journal.pone.0300760)
Supplement: S1 Table — (DOCX) [file pone.0300760.s001.docx]

S1 Table. List of genotypes used in the study.

| **Sr.#** | **Genotype** | **Dose** | **Parent** | **Pedigree** | **Generation** |
| --- | --- | --- | --- | --- | --- |
| 1 | Mu-AS-1 | 150 | RICF-160 | CH-1847-15-1 | M4 |
| 2 | Mu-AS-2 | 150 | RICF-160 | CH-1847-15-2 | M4 |
| 3 | Mu-AS-3 | 150 | RICF-160 | CH-1847-15-3 | M4 |
| 4 | Mu-AS-4 | 150 | RICF-160 | CH-1847-15-4 | M4 |
| 5 | Mu-AS-5 | 150 | RICF-160 | CH-1847-15-5 | M4 |
| 6 | Mu-AS-6 | 150 | RICF-160 | CH-1847-15-6 | M4 |
| 7 | Mu-AS-7 | 150 | RICF-160 | CH-1847-15-7 | M4 |
| 8 | Mu-AS-8 | 150 | RICF-160 | CH-1847-15-8 | M4 |
| 9 | Mu-AS-9 | 150 | RICF-160 | CH-1847-15-9 | M4 |
| 10 | Mu-AS-10 | 150 | RICF-160 | CH-1847-15-10 | M4 |
| 11 | Mu-AS-11 | 150 | RICF-160 | CH-1847-15-11 | M4 |
| 12 | Mu-AS-12 | 150 | RICF-160 | CH-1847-15-12 | M4 |
| 13 | Mu-AS-13 | 150 | RICF-160 | CH-1847-15-13 | M4 |
| 14 | Mu-AS-14 | 150 | RICF-160 | CH-1847-15-14 | M4 |
| 15 | Mu-AS-15 | 150 | RICF-160 | CH-1847-15-15 | M4 |
| 16 | Mu-AS-16 | 200 | RICF-160 | CH-1847-20-1 | M4 |
| 17 | Mu-AS-17 | 200 | RICF-160 | CH-1847-20-2 | M4 |
| 18 | Mu-AS-18 | 200 | RICF-160 | CH-1847-20-3 | M4 |
| 19 | Mu-AS-19 | 200 | RICF-160 | CH-1847-20-4 | M4 |
| 20 | Mu-AS-20 | 200 | RICF-160 | CH-1847-20-5 | M4 |
| 21 | Mu-AS-21 | 200 | RICF-160 | CH-1847-20-6 | M4 |
| 22 | Mu-AS-22 | 200 | RICF-160 | CH-1847-20-7 | M4 |
| 23 | Mu-AS-23 | 200 | RICF-160 | CH-1847-20-8 | M4 |
| 24 | Mu-AS-24 | 200 | RICF-160 | CH-1847-20-9 | M4 |
| 25 | Mu-AS-25 | 200 | RICF-160 | CH-1847-20-10 | M4 |
| 26 | Mu-AS-26 | 200 | RICF-160 | CH-1847-20-11 | M4 |
| 27 | Mu-AS-27 | 200 | RICF-160 | CH-1847-20-12 | M4 |
| 28 | Mu-AS-28 | 200 | RICF-160 | CH-1847-20-13 | M4 |
| 29 | Mu-AS-29 | 200 | RICF-160 | CH-1847-20-14 | M4 |
| 30 | Mu-AS-30 | 200 | RICF-160 | CH-1847-20-15 | M4 |
| 31 | Mu-AS-31 | 200 | RICF-160 | CH-1847-20-16 | M4 |
| 32 | Mu-AS-32 | 250 | RICF-160 | CH-1847-25-1 | M4 |
| 33 | Mu-AS-33 | 250 | RICF-160 | CH-1847-25-2 | M4 |
| 34 | Mu-AS-34 | 250 | RICF-160 | CH-1847-25-3 | M4 |
| 35 | Mu-AS-35 | 250 | RICF-160 | CH-1847-25-4 | M4 |
| 36 | Mu-AS-36 | 250 | RICF-160 | CH-1847-25-5 | M4 |
| 37 | Mu-AS-37 | 250 | RICF-160 | CH-1847-25-6 | M4 |
| 38 | Mu-AS-38 | 250 | RICF-160 | CH-1847-25-7 | M4 |
| 39 | Mu-AS-39 | 250 | RICF-160 | CH-1847-25-8 | M4 |
| 40 | Mu-AS-40 | 250 | RICF-160 | CH-1847-25-9 | M4 |
| 41 | Mu-AS-41 | 250 | RICF-160 | CH-1847-25-10 | M4 |
| 42 | Mu-AS-42 | 250 | RICF-160 | CH-1847-25-11 | M4 |
| 43 | Mu-AS-43 | 250 | RICF-160 | CH-1847-25-12 | M4 |
| 44 | Mu-AS-44 | 250 | RICF-160 | CH-1847-25-13 | M4 |
| 45 | Mu-AS-45 | 250 | RICF-160 | CH-1847-25-14 | M4 |
| 46 | Mu-AS-46 | 250 | RICF-160 | CH-1847-25-15 | M4 |
| 47 | Mu-AS-47 | 250 | RICF-160 | CH-1847-25-16 | M4 |
| 48 | Hy-AS-48 | Hybrid | RICF-160/ELD | DRM-32-1 | F6 |
| 49 | Hy-AS-49 | Hybrid | RICF-160/ELD | DRM-32-2 | F6 |
| 50 | Hy-AS-50 | Hybrid | RICF-160/ELD | DRM-32-3 | F6 |
| 51 | Hy-AS-51 | Hybrid | RICF-160/ELD | DRM-32-4 | F6 |
| 52 | Hy-AS-52 | Hybrid | RICF-160/ELD | DRM-32-5 | F6 |
| 53 | Hy-AS-53 | Hybrid | RICF-160/ELD | DRM-32-6 | F6 |
| 54 | Hy-AS-54 | Hybrid | RICF-160/ELD | DRM-32-7 | F6 |
| 55 | Hy-AS-55 | Hybrid | RICF-160/ELD | DRM-32-8 | F6 |
| 56 | Hy-AS-56 | Hybrid | RICF-160/ELD | DRM-32-9 | F6 |
| 57 | Hy-AS-57 | Hybrid | RICF-160/ELD | DRM-32-10 | F6 |
| 58 | Hy-AS-58 | Hybrid | RICF-160/ELD | DRM-32-11 | F6 |
| 59 | Hy-AS-59 | Hybrid | RICF-160/ELD | DRM-32-12 | F6 |
| 60 | Hy-AS-60 | Hybrid | RICF-160/ELD | DRM-32-13 | F6 |
| 61 | Hy-AS-61 | Hybrid | RICF-160/ELD | DRM-32-14 | F6 |
| 62 | Hy-AS-62 | Hybrid | RICF-160/ELD | DRM-32-15 | F6 |
| 63 | Hy-AS-63 | Hybrid | RICF-160/ELD | DRM-32-16 | F6 |
| 64 | Hy-AS-64 | Hybrid | RICF-160/ELD | DRM-32-17 | F6 |
| 65 | Hy-AS-65 | Hybrid | RICF-160/ELD | DRM-32-18 | F6 |
| 66 | Hy-AS-66 | Hybrid | RICF-160/ELD | DRM-32-19 | F6 |
| 67 | Hy-AS-67 | Hybrid | RICF-160/ELD | DRM-32-20 | F6 |
| 68 | Hy-AS-68 | Hybrid | RICF-160/ELD | DRM-32-21 | F6 |
| 69 | Hy-AS-69 | Hybrid | RICF-160/ELD | DRM-32-22 | F6 |
| 70 | Hy-AS-70 | Hybrid | RICF-160/ELD | DRM-32-23 | F6 |
| 71 | Hy-AS-71 | Hybrid | RICF-160/ELD | DRM-32-24 | F6 |
| 72 | Hy-AS-72 | Hybrid | RICF-160/ELD | DRM-32-25 | F6 |
| 73 | Hy-AS-73 | Hybrid | RICF-160/ELD | DRM-32-26 | F6 |
| 74 | Hy-AS-74 | Hybrid | RICF-160/ELD | DRM-32-27 | F6 |
| 75 | Hy-AS-75 | Hybrid | RICF-160/ELD | DRM-32-28 | F6 |
| 76 | Hy-AS-76 | Hybrid | RICF-160/ELD | DRM-32-29 | F6 |
| 77 | Hy-AS-77 | Hybrid | RICF-160/ELD | DRM-32-30 | F6 |
| 78 | Hy-AS-78 | Hybrid | RICF-160/ELD | DRM-32-31 | F6 |
| 79 | Hy-AS-79 | Hybrid | RICF-160/ELD | DRM-32-32 | F6 |
| 80 | Hy-AS-80 | Hybrid | RICF-160/ELD | DRM-32-33 | F6 |
| 81 | Hy-AS-81 | Hybrid | RICF-160/ELD | DRM-32-34 | F6 |
| 82 | Hy-AS-82 | Hybrid | RICF-160/ELD | DRM-32-35 | F6 |
| 83 | Hy-AS-83 | Hybrid | RICF-160/ELD | DRM-32-36 | F6 |
| 84 | Hy-AS-84 | Hybrid | RICF-160/ELD | DRM-32-37 | F6 |
| 85 | Hy-AS-85 | Hybrid | RICF-160/ELD | DRM-32-38 | F6 |
| 86 | Hy-AS-86 | Hybrid | RICF-160/ELD | DRM-32-39 | F6 |
| 87 | Hy-AS-87 | Hybrid | RICF-160/ELD | DRM-37-1 | F6 |
| 88 | Hy-AS-88 | Hybrid | RICF-160/ELD | DRM-37-2 | F6 |
| 89 | Hy-AS-89 | Hybrid | RICF-160/ELD | DRM-37-3 | F6 |
| 90 | Hy-AS-90 | Hybrid | RICF-160/ELD | DRM-37-4 | F6 |
| 91 | Hy-AS-91 | Hybrid | RICF-160/ELD | DRM-37-5 | F6 |
| 92 | Hy-AS-92 | Hybrid | RICF-160/ELD | DRM-37-6 | F6 |
| 93 | Hy-AS-93 | Hybrid | RICF-160/ELD | DRM-37-7 | F6 |
| 94 | Hy-AS-94 | Hybrid | RICF-160/ELD | DRM-37-8 | F6 |
| 95 | Hy-AS-95 | Hybrid | RICF-160/ELD | DRM-37-9 | F6 |
| 96 | Hy-AS-96 | Hybrid | RICF-160/ELD | DRM-37-10 | F6 |
| 97 | Hy-AS-97 | Hybrid | RICF-160/ELD | DRM-37-11 | F6 |
| 98 | Hy-AS-98 | Hybrid | RICF-160/ELD | DRM-37-12 | F6 |
| 99 | Hy-AS-99 | Hybrid | RICF-160/ELD | DRM-37-13 | F6 |
| 100 | Hy-AS-100 | Hybrid | RICF-160/ELD | DRM-37-14 | F6 |
| 101 | Hy-AS-101 | Hybrid | RICF-160/ELD | DRM-38--1 | F6 |
| 102 | Hy-AS-102 | Hybrid | RICF-160/ELD | DRM-38--2 | F6 |
| 103 | Hy-AS-103 | Hybrid | RICF-160/ELD | DRM-38--3 | F6 |
| 104 | Hy-AS-104 | Hybrid | RICF-160/ELD | DRM-38--4 | F6 |
| 105 | Hy-AS-105 | Hybrid | RICF-160/ELD | DRM-38--5 | F6 |
| 106 | Hy-AS-106 | Hybrid | RICF-160/ELD | DRM-38--6 | F6 |
| 107 | Hy-AS-64 | Hybrid | RICF-160/ELD | DRM-38--7 | F6 |
| 108  109 | Super Basmati  RICF-160 |  |  |  |  |
