# Supplementary material for: Identification of biochemical indices for brown spot (Bipolaris oryzae) disease resistance in rice mutants and hybrids
Source: PLoS One. 2024 Apr 18;19(4):e0300760. doi: 10.1371/journal.pone.0300760 (PMC11025958; doi:10.1371/journal.pone.0300760)
Supplement: S2 Table — (DOCX) [file pone.0300760.s002.docx]

S2 Table. List of genotypes used in biochemical study.

| **7** | **Genotype** | **Dose** | **Parent** | **Pedigree** | **Generation** |
| --- | --- | --- | --- | --- | --- |
| 1 | Hy-AS-92 | Hybrid | RICF-160/ELD | DRM-37-6 | F6 |
| 2 | Hy-AS-98 | Hybrid | RICF-160/ELD | DRM-37-12 | F6 |
| 3 | Hy-AS-99 | Hybrid | RICF-160/ELD | DRM-37-13 | F6 |
| 4 | Hy-AS-101 | Hybrid | RICF-160/ELD | DRM-38--1 | F6 |
| 5 | Hy-AS-102 | Hybrid | RICF-160/ELD | DRM-38--2 | F6 |
| 6 | Hy-AS-107 | Hybrid | RICF-160/ELD | DRM-38--7 | F6 |
| 7 | Mu-AS-2 | 150 | RICF-160 | CH-1847-15-2 | M4 |
| 8 | Mu-AS-22 | 200 | RICF-160 | CH-1847-20-7 | M4 |
| 9 | Mu-AS-37 | 250 | RICF-160 | CH-1847-25-6 | M4 |
| 10 | Mu-AS-40 | 250 | RICF-160 | CH-1847-25-9 | M4 |
| 11 | Hy-AS-94 | Hybrid | RICF-160/ELD | DRM-37-8 | F6 |
| 12 | Hy-AS-105 | Hybrid | RICF-160/ELD | DRM-38--5 | F6 |
| 13 | Mu-AS-28 | 200 | RICF-160 | CH-1847-20-13 | M4 |
| 14 | Mu-AS-8 | 150 | RICF-160 | CH-1847-15-8 | M4 |
| 15 | Hy-AS-103 | Hybrid | RICF-160/ELD | DRM-38--3 | F6 |
| 16 | Mu-AS-19 | 200 | RICF-160 | CH-1847-20-4 | M4 |
| 17 | Mu-AS-20 | 200 | RICF-160 | CH-1847-20-5 | M4 |
| 18 | Mu-AS-32 | 250 | RICF-160 | CH-1847-25-1 | M4 |
| 19 | Mu-AS-35 | 250 | RICF-160 | CH-1847-25-4 | M4 |
| 20 | Mu-AS-42 | 250 | RICF-160 | CH-1847-25-11 | M4 |
| 21 | Mu-AS-44 | 250 | RICF-160 | CH-1847-25-13 | M4 |
| 22 | Hy-AS-57 | Hybrid | RICF-160/ELD | DRM-32-10 | F6 |
| 23 | Mu-AS-7 | 150 | RICF-160 | CH-1847-15-7 | M4 |
| 24 | Mu-AS-43 | 250 | RICF-160 | CH-1847-25-12 | M4 |
| 25 | Mu-AS-18 | 200 | RICF-160 | CH-1847-20-3 | M4 |
| 26 | Mu-AS-47 | 250 | RICF-160 | CH-1847-25-16 | M4 |
| 27 | Mu-AS-1 | 150 | RICF-160 | CH-1847-15-1 | M4 |
| 28 | Mu-AS-25 | 200 | RICF-160 | CH-1847-20-10 | M4 |
| 29 | Hy-AS-54 | Hybrid | RICF-160/ELD | DRM-32-7 | F6 |
| 30 | Mu-AS-21 | 200 | RICF-160 | CH-1847-20-6 | M4 |
| 31 | Hy-AS-55 | Hybrid | RICF-160/ELD | DRM-32-8 | F6 |
| 32 | Mu-AS-6 | 150 | RICF-160 | CH-1847-15-6 | M4 |
| 33 | Hy-AS-67 | Hybrid | RICF-160/ELD | DRM-32-20 | F6 |
| 34 | Mu-AS-12 | 150 | RICF-160 | CH-1847-15-12 | M4 |
| 35 | Mu-AS-10 | 150 | RICF-160 | CH-1847-15-10 | M4 |
| 36 | Mu-AS-15 | 150 | RICF-160 | CH-1847-15-15 | M4 |
| 37 | Hy-AS-63 | Hybrid | RICF-160/ELD | DRM-32-16 | F6 |
| 38 | Hy-AS-50 | Hybrid | RICF-160/ELD | DRM-32-3 | F6 |
| 39 | Hy-AS-60 | Hybrid | RICF-160/ELD | DRM-32-13 | F6 |
| 40 | Hy-AS-51 | Hybrid | RICF-160/ELD | DRM-32-4 | F6 |
| 41 | Hy-AS-56 | Hybrid | RICF-160/ELD | DRM-32-9 | F6 |
| 42 | Hy-AS-58 | Hybrid | RICF-160/ELD | DRM-32-11 | F6 |
| 43 | Hy-AS-64 | Hybrid | RICF-160/ELD | DRM-32-17 | F6 |
| 44  45 | SuperBasmati  RICF-160 |  |  |  |  |
